# Defense Responses in Two Ecotypes of *Lotus japonicus* against Non-Pathogenic *Pseudomonas syringae*

**DOI:** 10.1371/journal.pone.0083199

**Published:** 2013-12-11

**Authors:** Cesar D. Bordenave, Francisco J. Escaray, Ana B. Menendez, Eva Serna, Pedro Carrasco, Oscar A. Ruiz, Andrés Gárriz

**Affiliations:** 1 Instituto de Investigaciones Biotecnológicas-Instituto Tecnológico de Chascomús/Universidad Nacional de General San Martín-Consejo Nacional de Investigaciones Científicas y Técnicas (IIB-INTECH/UNSAM-CONICET), Chascomús, Argentina; 2 Unidad Central de Investigación en Medicina-INCLIVA, Universitat de Valencia, Valencia, Spain; 3 Departamento de Bioquímica y Biología Vegetal-Universitat de Valencia, Valencia, Spain; Agriculture and Agri-Food Canada, Canada

## Abstract

*Lotus japonicus* is a model legume broadly used to study many important processes as nitrogen fixing nodule formation and adaptation to salt stress. However, no studies on the defense responses occurring in this species against invading microorganisms have been carried out at the present. Understanding how this model plant protects itself against pathogens will certainly help to develop more tolerant cultivars in economically important *Lotus* species as well as in other legumes. In order to uncover the most important defense mechanisms activated upon bacterial attack, we explored in this work the main responses occurring in the phenotypically contrasting ecotypes MG-20 and Gifu B-129 of *L. japonicus* after inoculation with *Pseudomonas syringae* DC3000 pv. tomato. Our analysis demonstrated that this bacterial strain is unable to cause disease in these accessions, even though the defense mechanisms triggered in these ecotypes might differ. Thus, disease tolerance in MG-20 was characterized by bacterial multiplication, chlorosis and desiccation at the infiltrated tissues. In turn, Gifu B-129 plants did not show any symptom at all and were completely successful in restricting bacterial growth. We performed a microarray based analysis of these responses and determined the regulation of several genes that could play important roles in plant defense. Interestingly, we were also able to identify a set of defense genes with a relative high expression in Gifu B-129 plants under non-stress conditions, what could explain its higher tolerance. The participation of these genes in plant defense is discussed. Our results position the *L. japonicus-P. syringae* interaction as a interesting model to study defense mechanisms in legume species.

## Introduction

Legume plants (*Fabaceae*) have accompanied mankind since the dawn of history, mainly due to the simplicity of their domestication and excellent nutritional values for humans and cattle [1]. In addition, their notable ability to establish symbiotic interactions with nitrogen-fixing bacteria, collectively known as rhizobia, constitutes a key feature for agricultural sustainability, as the nitrogen incorporated by this mean reduces the costs derived from the use of fertilizers and raises soil nutrient contents [2]. 

The utilization of legumes, in particular soybean, has been boosted in the last decades. This was allowed by the introduction of genetically improved crops as well as new plant varieties with higher adaptability to constrained lands [1,3]. Nevertheless, legume production is still hampered in many areas by various abiotic stresses as drought, high salinity and soil nutrient depletion, in addition to biotic stress originated by pathogenic microorganisms. This last factor is the cause of considerable losses every year [1], a situation that could be worsen over the near future as long as the current expansion in legume utilization continues. Therefore, a deeper understanding of the defense responses deployed by legume plants against pathogens is a crucial step in the development of tolerant cultivars and the establishment of effective disease control strategies. 

Plant resistance against pathogens entails a reprogramming in gene expression [4], where reactive oxygen species (ROS), as well as plant hormones like salicylic acid, jasmonic acid and ethylene play important roles. These compounds activate and amplify the defense responses through a complex network of transcription factors [5]. Nowadays, a vast research has been carried out on the defense mechanisms deployed under these conditions in model plants as *Arabidopsis*, tobacco and tomato [6–8]. However, there is a rather lack of information on the defense responses that control attacking pathogens in legumes [9]. To make this even worse, the resistance mechanisms identified in plants belonging to other families cannot be fully extrapolated to legume species, probably due to differences in genomic organization [10]. Thus, how legumes defend themselves from invading microorganisms is an area that deserves more attention if pathogen resistance in economically important legume species wants to be enhanced. 

Fortunately, the advent in the last years of feasible microarray platforms developed in model legume species has prompted more detailed explorations on this topic. Thus, transcriptomic analyses were addressed to characterize plant–pathogen interactions in *Medicago truncatula* [11,12] and *Glycine max* [13]. These reports identified a pool of genes whose expression is regulated specifically in resistant genotypes during pathogenic interactions (which of course, should be considered as interesting targets for further studies). In addition, these works also revealed that many genes are regulated in a similar way in resistant and susceptible plants. This last observation suggests that the success of the defense is not only explained by the regulation of particular genes in resistant materials, but the extent and time of transcriptional reprogramming of a collection of genes whose expression is ultimately modulated in all genotypes. For instance, a large set of genes is equally regulated in *G. max* against *Pseudomonas syringae* pv. *glycinea* expressing or lacking the avirulence gene *avrB* [13]. However, the plant is only resistant to the disease in the first case, a phenomenon associated to a higher degree of gene expression regulation. A similar conclusion may be drawn from the studies on other non-legume plant species [14].

In the last years, *Lotus japonicus* has been adopted by the scientific community as a model species for legume research. It offers all the properties shown by other classical models, that is, small genome size, self-fertility and a short life cycle, with the addition of some biological differences with other legumes models what make it, at some extent, an unique representative among this group [15,16]. For instance, this species presents perennial growth and determinate nodulation, in contrast to annual growth and indeterminate nodulation in *M. truncatula*. Recently, many genetic resources were developed in *L. japonicus* and have played a determinant role in the progress achieved on legume research, particularly on subjects as symbiosis development, and long-salt stress acclimatization [17–20]. 

In order to add more light to the current knowledge on legume defense responses to invading microorganisms, in this report we examined the interaction between *L. japonicus* and *P. syringae* pv. tomato DC3000 (*Pto*), a strain that cause bacterial speck in tomato and *Arabidopsis* [21]. Importantly, some of the genes associated to virulence in this strain diverge from those described in other legume-parasitic races of this species, as pv. *glycinea*, pv. *phaseolicola* and pv. *pisi*. Based on this, we speculated that *L. japonicus* defense responses would be successful in restricting bacterial leaf colonization. Thus, these partners could be developed into a useful model pathosystem to study the most general defense mechanisms deployed in this legume against non-pathogenic microorganisms. We envision that this type of studies will complement our future analysis of the *L. japonicus* interaction with legume-infecting *P. syringae* pathovars. 

In this work, we first conducted a phenotypic characterization of the interaction between *Pto* and two of the most widely used *L. japonicus* genotypes, Gifu B-129 and Miyakojima MG-20. Interestingly, our analysis demonstrated the existence of quite contrasting phenotypic differences in the two ecotypes during the response to the bacteria. On these grounds, we next performed a transcriptomic analysis aimed to identify the genes associated with such differential response and decipher the main defense mechanisms that occur in this plant species. We were able to recognize a large number of transcripts differentially expressed, many of them showing high homology to well-known defense genes in other plant species. These genes and their putative function on plant defense are discussed. 

## Materials and Methods

### Biological material and growth conditions

 Seeds of *L. japonicus* MG-20 and Gifu B-129 were treated with concentrated sulfuric acid for 2 min., rinsed ten times with sterile distilled water and germinated in Petri dishes containing agar/water (0.8%). Seedlings were transferred to sand-perlite soil mix (1:1) and cultivated in growth chamber with a 16h day/8h night photoperiod (photon flux density of 200 μmol m s^–1^ provided by cool-white fluorescent lamps) and 24/21 ± 2°C and 55/ 65 ± 5% day/night temperature and relative humidity, respectively. Plants were regularly irrigated with half-strength Hoagland’s nutrient solution [22]. Three week-old *L. japonicus* plants (with 6 to 8 true leaves) were used in all experiments.

*Pto* inocula were prepared by growing bacteria in King’s B agar-medium [23] plates at 28°C for 48 h and scraping cells off in 10 mM MgCl_2_, pH 7.0, to a cell density of 0.1 A_600_.

### Bacterial counts and ROS detection

In order to assess bacterial growth in *L. japonicus* ecotypes, four central leaflets per plant were infiltrated with a bacterial suspension using a needleless 1-ml syringe, as described by Katagiri et al. [24]. Mocked-inoculated controls were infiltrated with 10 mM MgCl_2,_ pH 7. Infiltrated leaflets were harvested 24, 48 or 72 hours post inoculation (hpi). Each biological replicate represents 4 pooled central leaflets from the same plant, and 6 different plants were used per treatment. Then, leaflets were weighted, surface sterilized in 70% ethanol by 30 sec, washed in sterile distilled water by another 30 sec and macerated with a microfuge tube pestle in 10 mM MgCl_2_, pH 7.0. After grinding the tissue, samples were thoroughly vortexed and serially diluted 1:10 in 10 mM MgCl_2_. Bacterial counts on infiltrated leaves were performed by plating 20 µl of these dilutions onto King’s B agar medium supplemented with rifampicin 50 µg/ml. Plates were placed at 28°C and colony-forming units counted after a period of 48 h. Each experiment was conducted three times with similar results. Data were analyzed by one-way ANOVA with Bonferroni’s post-hoc tests.

ROS accumulation was evaluated on detached central leaflets. First, petioles were submerged in a 15 μM solution of the redox-sensitive dye 2’,7’-dichlorofluorescein diacetate (DCFDA) for 30 min. Then, central leaflets were infiltrated with the bacterial solution and the petioles submerged in fresh DCFDA solution. Green fluorescence was visualized by epifluorescence (excitation filter, 460 nm; emission filter, >515 nm) at 1, 6 and 24 h after infiltration. Treatments were set up in triplicate, and each experiment was conducted three times with similar results. 

### RNA extraction and microarray-based transcriptomic analysis

For total RNA extraction, 4 leaflets per plant were harvested at 24 hpi (inoculated with *Pto* or MgCl_2_), each treatment consisting of 24 plants. Leaflets from all 24 plants with the same treatment were pooled, frozen in liquid nitrogen and stored at -80°C. This pool represented one biological replicate. Overall, there were 3 biological replicates corresponding to 3 independent experiments performed with a 2-week interval. Total RNA was extracted from frozen tissues using Spectrum^TM^ Plant Total RNA kit (Sigma-Aldrich) according to the manufacturer’s instructions. Prior to microarray analyses, the integrity of the RNA was checked on agarose gels. RNA (300 ng/sample) was then amplificated and labeled using the GeneChip® 3´IVT Express kit (Affymetrix) as described by the manufacturer. All biological replicates were independently hybridized to GeneChip® *Lotus japonicus* custom (Affymetrix) containing more than 50,000 probesets, each representing a known or predicted plant gene sequence (Affymetrix; http://www.affymetrix.com). Arrays were scanned on an Affymetrix GeneChip® Scanner 3000 7G and the GeneChip Operating Software was used to perform gene expression analysis. Data (.CEL files) were analyzed and statistically filtered using the Robin software [25]. Input files were normalized with the RMA algorithm and statistically significant genes were identified using ANOVA-1 way model analysis of variance with a false discovery rate (FDR correction) of *P*<0.01. We used a ± 2-fold change in gene expression as the cut-off value. Functional classification of significantly regulated genes was conducted importing expression data into the MAPMAN software after converting fold change values to log_2_ in Excel files, as described by Thimm et al [26] and later updated by Usadel et al [27]. 

The data (.CEL files) presented in this publication have been deposited in the ArrayExpress database (http://www.ebi.ac.uk/arrayexpress/) and are accessible through the accession number E-MTAB-2000.

### Quantitative RT-PCR

To validate microarray values by quantitative real time PCR, 2.5 µl from a tenfold dilution of the cDNA stock was further diluted to 15 µl with primer mix (300 nM final concentration), 7.5µl of FastStart Universal SYBR Green Master (Rox) and the required amount of double distilled water. Primers used in these reactions are listed in Table 1. Reactions were performed in an Mx3005P qPCR System with the help of the MxPro qPCR Software 4.0 (Stratagene®, La Jolla, CA, U.S.A.). Relative quantification was performed by the comparative cycle threshold method using the INFOSTAT (InfoStat, 2008. InfoStat Group, FCA, Universidad Nacional de Cordoba, Argentina) with elongation factor 1α gene (EF-1α) as endogenous control [28]. For comparative purposes, relative gene expression in control plants was defined as 1. 

**Table 1:** Primers used in this work.

**Gen ID**	**Probe set ID**	**Primer sequence**	
		**Forward**	**Reverse**
chr1.ljb18k24.110.r2.a	chr1.bm1732.2_at	ATGCCTCCTTCACTTAGG	CTTCACTATATTCAGAGATCACTT
chr3.cm0091.1150.r2.d	chr3.cm0216.2_at	GGAGGCAAGCGGAGATATAC	CGGTCCACATCAAATCCAAAC
chr3.cm0279.1210.r2.d	chr3.cm0279.2_at	TGGAGGTCATAGTAGTATCT	GAGGACTCACTTCTTCAT
chr5.cm0062.220.r2.d	chr5.cm0062.23_at	GCTATCTCGTGTTCAAGG	CTATCAGTGCTATCATAAGTTG
chr5.cm0344.440.r2.d	chr5.cm0344.11_at	GGCTTAACAACAATAGACTGAG	AACTTATAGGAGTGAATAATGCG
chr5.cm0200.390.r2.d	chr5.cm0953.1_at	TGAATGATGAAATGCCTAAGAG	CTTCTCCACCACTCCATT
chr5.LjT17N18.60.r2.d	chr5.tm1493.8_at	CTGGTGGTAATGAAGTCAAC	GAACTCTGCCAACTCTCG
chr6.cm0139.1430.r2.d	chr6.cm0539.8_at	CTTCCACAACTATGACAT	AACACAACATTATACTCCTT
chr4.cm0528.420.r2.d	cm0528.2_at	CTCGTCAAACAACTTCAC	CAATGGCACAAATCCTAAA
chr3.cm279.180.r2.d/130.r2.d	ljwgs_011581.2_at	AAGTTGTCATCCAAGTTG	GTAGTAGTTCATATTCACCAT
LjSGA_013445.2	ljwgs_013445.2_at	GGGTTTGGAGACCATTAGAA	GCACACACTGGGACAATA
chr4.cm1622.120.r2.d	ljwgs_020594.1_at	ATGCCATCCAGAGTGTTG	TATACCAAGTTAGCCTCATCTATT
chr1.LjT46A21.140.r2.a	ljwgs_023901.2_at	GCTACATTACCCTTCTTG	CCATTGCTCATTATCTCC
chr3.cm2163.270.r2.m	ljwgs_025651.1.1_at	CTCCTCTCACTCACTCTCACTCTC	TCTCTGTGCGTGGTTGTTGTTC
LjSGA_025735.1	ljwgs_025735.1_at	CCAAGTGATGTTACAGTTAC	TGTCCTCTGCTTCTATTATC
LjSGA_045519.1/LjSGA_061086.1.1	ljwgs_061086.1.1_at	GCATCAGATCCTAACGAC	CCCACAGAGAACTCAGAG
chr5.cm1077.690.r2.m	ljwgs_068360.1_at	CTCACACTTCTTCTCCAA	AAGGCATTGTTGATAGGT
chr1.cm0295.1210.r2.a/1190.r2.a	ljwgs_086126.1_at	GAGCACTTGAACATTGAA	TCCACTAACATCCTTGAG
chr6.cm0013.530.r2.m	ljwgs_090338.1_at	GCTATCACGCCAGACAAT	AACTCCTCCATTCCAGAAC
Fe1αEF (Housekeeping gene)		TGACAAGCGTGTGATCGAGAGG	GATACCTCTTTCACGCTCAGCCTT

## Results and Discussion

### Macroscopic symptoms and bacterial growth in infected leaves

**Figure 1:**
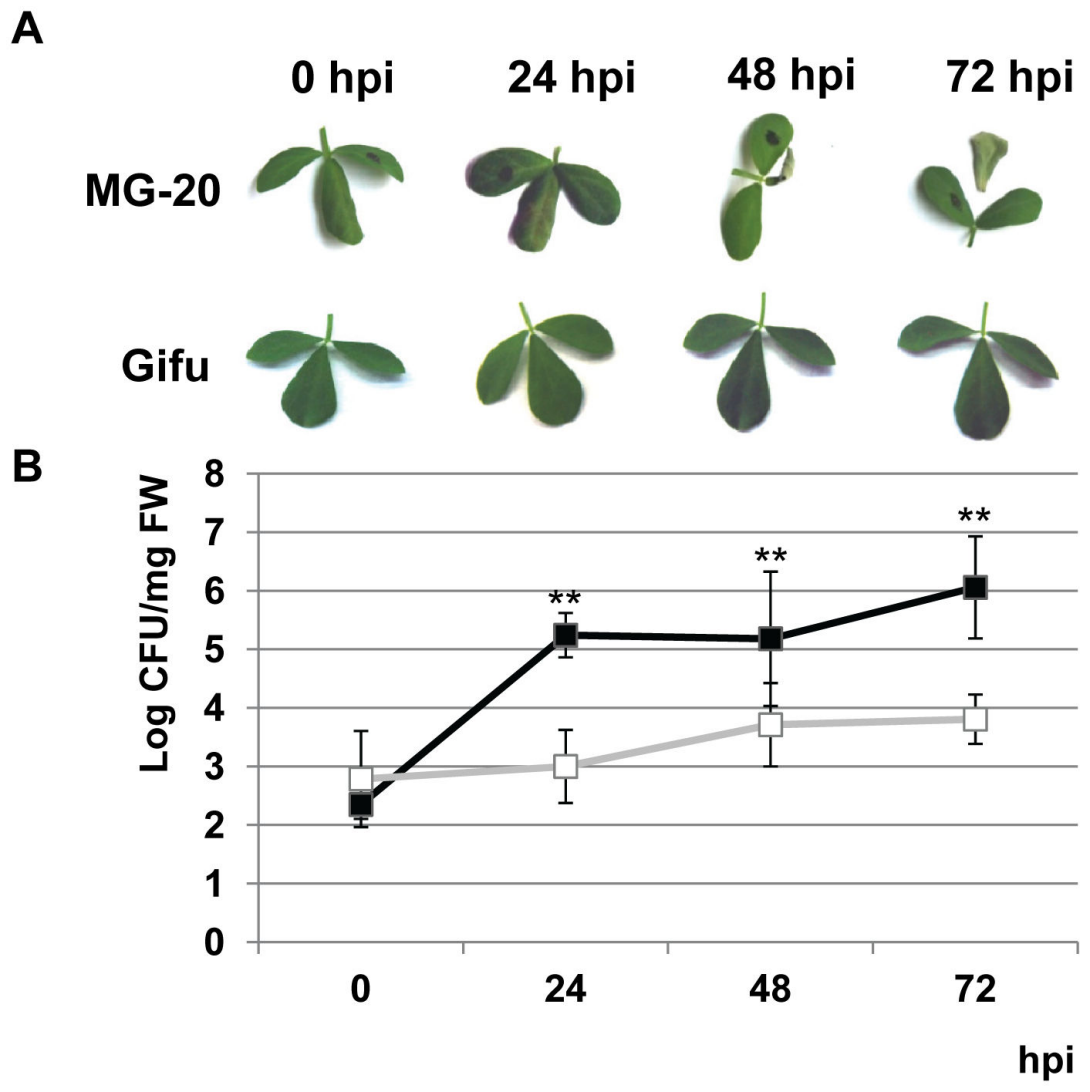
Differential phenotypes of *L. japonicus* ecotypes MG-20 and Gifu B-129 against *Pto*. **A**. Symptom development following bacterial infiltration in leaves from both ecotypes. Central leaflets were infiltrated using a needleless syringe as described in Materials and Methods. Eventually, lateral leaflets were marked with a black pen just for identification purposes. Pictures were taken immediately before inoculation and after a period of 24, 48 or 72 h. B. Measurement of *Pto* growth within MG-20 and Gifu B-129 infiltrated leaves. Data points represent the mean ± SD of three replicate experiments. Bacterial levels at 24, 48 and 72 hpi were statistical analyzed and compared against bacterial levels at 0 hpi, ** p<0.01. Black squares, MG-20; white squares, Gifu B-129.

*L. japonicus* accessions MG-20 and Gifu B-129 were compared with respect to their performance upon *Pto* inoculation. The first symptoms were observed in MG-20 at 24 h after bacterial infiltration. These symptoms were characterized by mild or partial dehydratation in the area surrounding the infiltration point. Leaf desiccation expanded thereafter, and occasionally small chlorotic spots were evident at 48 hpi, symptoms that continued aggravating during the course of the experiment (Figure 1A). The progress of the symptoms resemble those occurring in *Arabidopsis* inoculated with this bacterial strain [24]. At last, it was observed that in most of the cases the infiltrated leaflets frequently collapsed and drop from the trifoliate leaf after 72 hpi. Importantly, it should be noted that the described symptoms were evident only in infiltrated leaflets and never reached adjacent tissues (Figure 1A). Besides, the reproductive cycle of inoculated plants was indistinguishable from that shown by mock-treated plants (data not shown). By contrast, infiltrated leaflets from the Gifu B-129 ecotype remained green without symptoms of disease at any time point (Figure 1A). 

To assess the relationship between the levels of tissue damage and bacterial multiplication, we next monitored *Pto* growth in infiltrated leaves of both ecotypes. Figure 1B shows that the onset of symptoms development in MG-20 (24 hpi) was correlated to a significant 10^3^-fold rise in bacterial population. Following this increment, bacterial levels stabilized and remained unaltered throughout the time-course of the experiment. In turn, there was no change in bacterial count after the first 24 h in Gifu B-129 leaves, followed by a further slight but not significant increment, which did not exceed 10^1^-fold (Figure 1B). No bacteria were found in non-infiltrated adjacent leaflets, indicating that *Pto* is unable to colonize these tissues (data not shown). 

### ROS production upon bacterial infiltration

**Figure 2:**
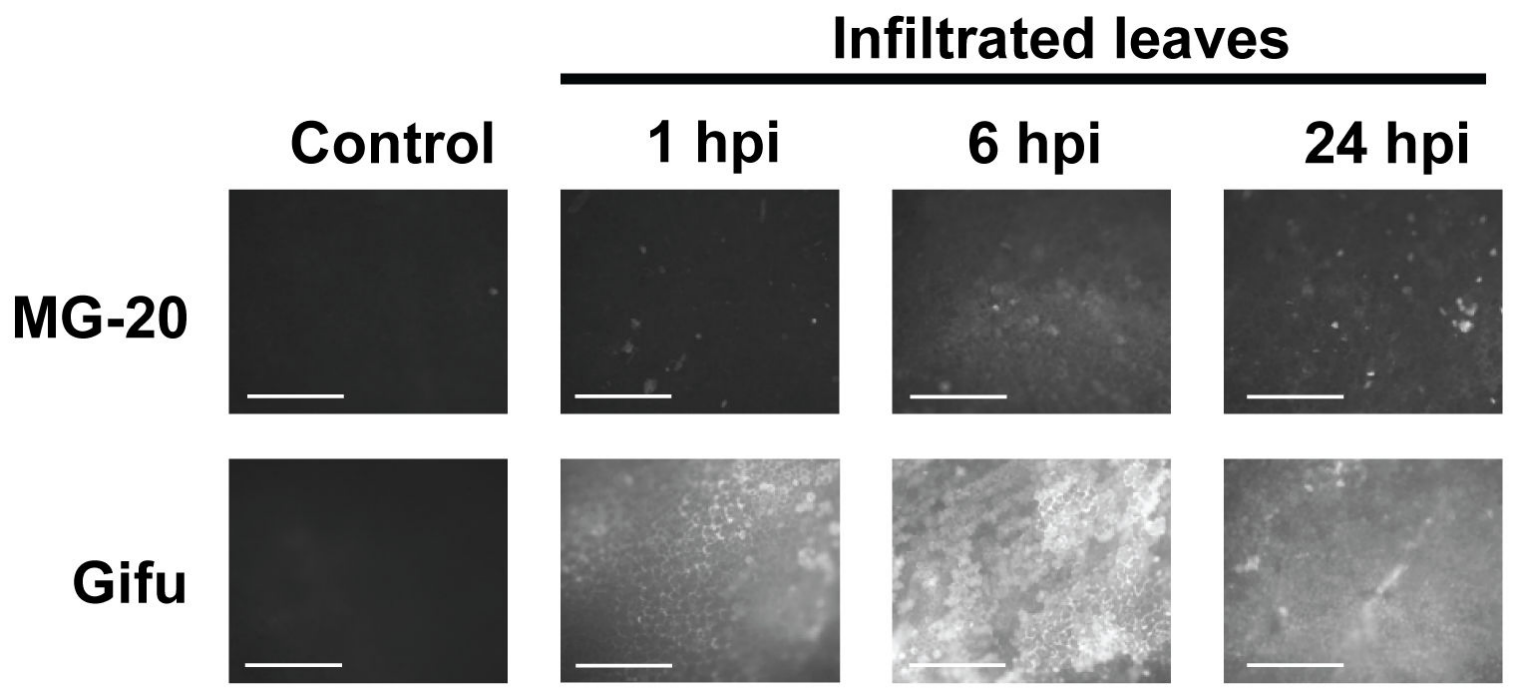
Effect of bacterial infiltration on ROS accumulation in *L. japonicus* leaves. Detached leaves of each ecotype were submerged for 30 min in a solution of 15 µM DCFDA and infiltrated with a bacterial solution or MgCl_2_ (controls). Then, leaves were incubated in freshly prepared DCFDA solution and observed under fluorescence microscopy at indicated time points (excitation filter, 460 nm; emission filter, >515 nm). Figure shows representative photographs of control and treated leaves. Bars: 500 µm.

In order to get a more profound characterization of the events that occur in the pathogenic interaction, we next evaluated the production of ROS in *L. japonicus* leaves challenged with *Pto*. The generation of these molecules is a key step during the early stages of pathogen recognition and plant defense activation [29]. With this purpose, petioles from detached leaves were submerged for 30 min in a 15 µM solution of the fluorescent dye DCFDA before inoculation, and observed by fluorescence microscopy after treatment at the indicated times. This analysis showed that a fast and dramatic burst in ROS production started in the Gifu B-129 ecotype as early as 1 hpi. The fluorescence signal reached then a maximal intensity at 6 hpi, and was slightly attenuated at 24 hpi (Figure 2). In turn, fluorescence in the MG-20 accession was undetectable until 6 hpi and it never reached the intensity observed in Gifu B-129. On other hand, ROS-derived fluorescence in Gifu B-129 appeared as large and diffuse areas spanning several cells, whereas small and intense spots (probably located at single cells) characterized the fluorescence evidenced in MG-20. Thus, not only the intensity of the signal and the time of its appearance, but also its location distinguished ROS generation in both ecotypes. 

Taken together, macroscopic observations and the evaluation of ROS production revealed a quite contrasting performance of the two ecotypes in response to bacterial inoculation. On one side, Gifu B-129 showed a high degree of tolerance to the bacteria. This is characterized by early production of ROS and the inability of the bacteria to grow in plant tissues, which correlate with the absence of symptoms. On the other hand, infiltrated leaves of MG-20 showed a relative delay in ROS generation, which is associated to a higher bacterial multiplication rate and the appearance of disease-like symptoms. Despite this, we cannot consider this ecotype as susceptible to the bacterial attack. This is based on the ability of the plant to block bacterial colonization of adjacent organs and restrict the damage only to infiltrated leaflets. Therefore, for further analysis we will consider that this ecotype shows a relative lower degree of tolerance compared to Gifu B-129. This means that the defense mechanisms involved in both ecotypes are not necessarily the same, and that some of the features that make Gifu B-129 able to suppress the growth of the pathogen and limit the negative effects derived from microorganism spreading are lacking in MG-20. Whether the responses associated to these phenotypes are preformed barriers or induced defenses (or both) will be discussed in the following paragraphs.

### Pto–induced transcript profiles in *L. japonicus* leaves

Gene expression in bacterial-inoculated leaves was compared to that in mock-treated controls from each ecotype using GeneChip® *Lotus japonicus* microarrays, which allows the simultaneous expression analysis of all known and predicted genes in *L. japonicus*. We selected samples taken 24 hpi since beyond that point MG-20 leaves were seriously damaged, what could mask changes in the expression of pathogen responding genes. This study revealed that bacterial infection altered transcriptional pattern in the two ecotypes, even though qualitative and quantitative differences were found between them. Thus, 5217 transcripts were regulated in MG-20, whereas only 458 were regulated in Gifu B-129 (Table S1). 

**Figure 3:**
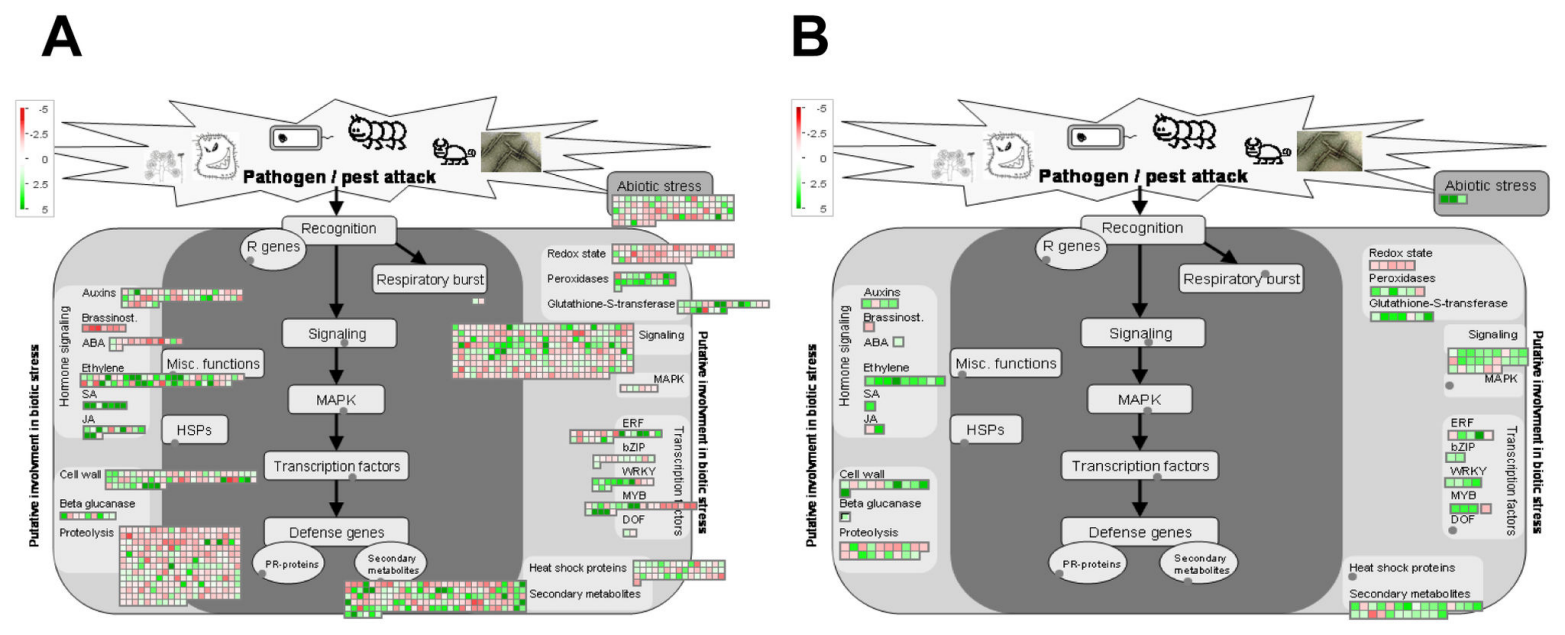
MapMan illustration depicting the transcripts from the “Biotic stress” BIN regulated under pathogenic conditions. Transcriptomic data from bacteria-inoculated leaves were compared to their respective controls (mock-inoculated). Genes that were shown to be differentially expressed were mapped using the MapMan software (http://mapman.gabipd.org). Picture shows genes related to biotic stress regulated in MG-20 (A) and Gifu (B). Log fold change ratios are indicated as a gradient of red (down-regulated) and green (up-regulated).

Nearly 43% of all the genes identified in MG-20 were up-regulated during the pathogenic process in comparison with mock-inoculated controls. Functional classification of both, up- and down-regulated genes by the MAPMAN software indicated a dramatic repression at several stages of photosynthesis (biosynthesis of photosystem components and tetrapyrrole) and primary metabolism, suggesting that different metabolic pathways other than energy assimilation are favored under this situation. In turn, 1189 out of the total genes regulated in this ecotype (22.8%) were classified as defense-related (Figure 3A). 

As stated above, a much lighter response in terms of gene expression regulation was observed to occur in Gifu B-129, since only 458 transcripts passed the applied filter criteria. However, the induced genes in this case reached a 76% of total regulated genes, a remarkably higher proportion than that determined in MG-20. The regulated genes in this ecotype were distributed in several functional groups, mainly those related to protein metabolism, RNA transcription regulation, secondary metabolism, and miscellaneous (peroxidases, acid phosphatases, cytochrome P450 family proteins, etc.). Around 29% of them (133 probesets) could be grouped within the “Biotic stress” category (Figure 3B). 

**Figure 4:**
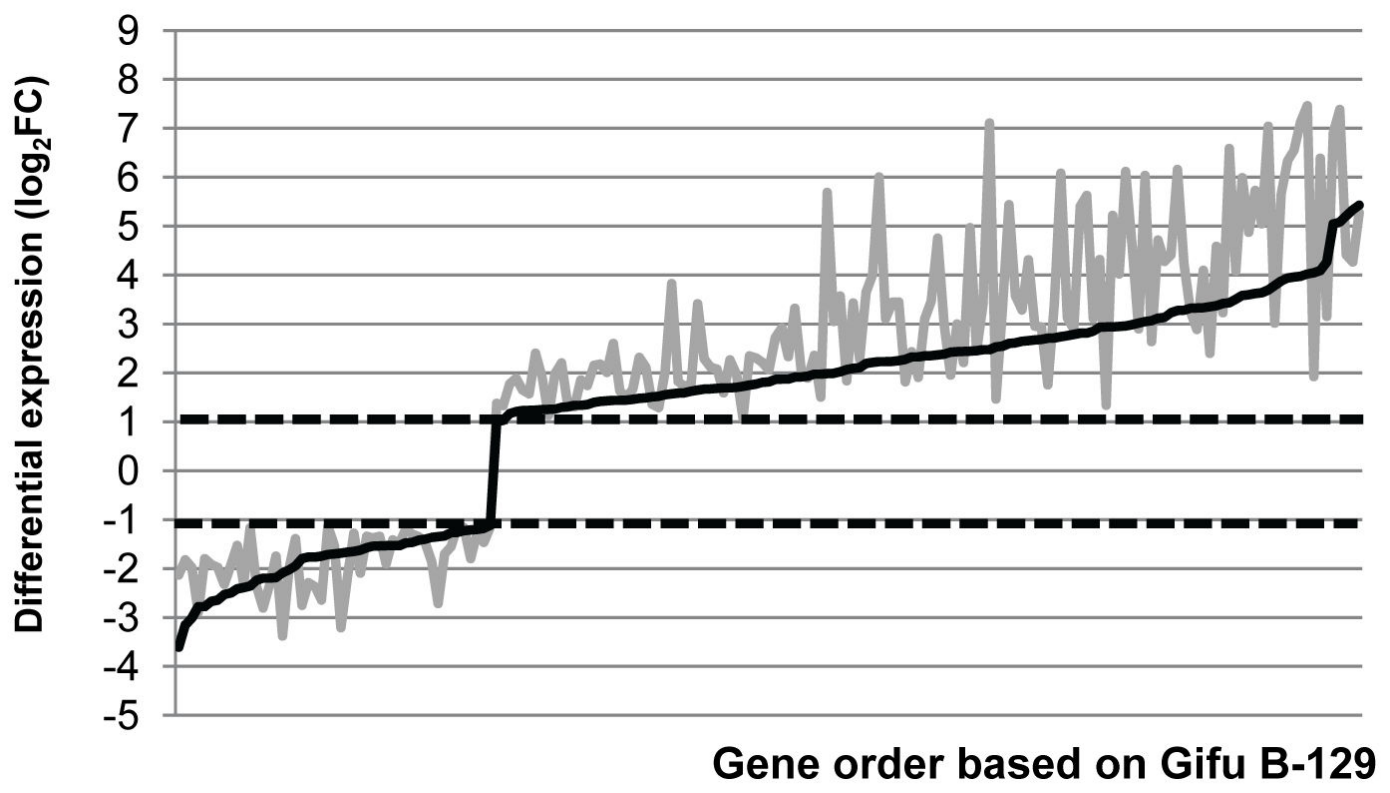
Trend plots comparing quantitative expression of differentially regulated genes in MG-20 and Gifu B-129. Gene order along the *x* axis was determined by the expression level in Gifu B-129 samples (dark lines). The expression of the same genes in MG-20 (grey lines) was plotted keeping the same order.

The full lists of genes regulated in each ecotype were then compared to each other. This examination demonstrated that 413 transcripts were regulated in MG-20 as well as in Gifu B-129. This amount includes the majority of the genes regulated in Gifu B-129 plants. Within this group, we sorted Gifu B-129 genes from the lowest fold-change to the highest and plotted them along with values for MG-20 (Figure 4). This graphical representation demonstrated that transcription regulation showed the same bias in both cases, suggesting that they comprehend a common repertoire of genes participating in general defense responses. However, we noticed that most of them were more strongly regulated in the case of MG-20. The probable participation of some of these genes in plant defense, as well as that of those regulated in only one ecotype is discussed in the following sections (genes considered are listed in Table S2).

### Genes related to pathogen perception and defense signaling

At present time, two major modes of pathogen perception are known in plants [30]. In the first mode, pathogen-associated molecular patterns (PAMPs) and damage-associated molecular patterns (DAMPs) are recognized by plant transmembrane pattern recognition receptors (PRRs), resulting in PAMP-triggered immunity (PTI) and DAMP-triggered immunity (DTI) [31]. In the second mode, the plant recognizes pathogens by using polymorphic nucleotide-binding site leucine-rich repeat (NB-LRR) proteins, encoded by cultivar-specific plant resistance (R) genes. This recognition ultimately leads to effector-triggered immunity (ETI). However, R genes trigger plant immunity when a matching effector, or avirulence protein (avr), is found in the pathogen. Therefore, this kind of defense is only effective against specific races of the same pathogen. In addition, it often (but not always) results in host cell death at the infection site, a process known as hypersensitive response (HR) [32]. PTI and ETI activate several defense mechanisms through a complex arrangement of signaling proteins. As *Pto* is not considered to cause diseases in legumes, we expect to find in our analysis components of the PAMP and DAMP responses rather that to ETI defense. 

In *Arabidopsis*, the leucine-rich repeat receptor-like kinases (LRR-RLKs) SERK3 and SERK4 function as signal enhancers of PAMP perception, by forming complexes with known PRRs as FLS2 and EFR1. In this trend, it has been demonstrated that SERK3 and SERK4 contribute to disease resistance against *Pto* [33]. Accordingly, we found that three SERK-like and one EFR-like transcripts were induced in MG-20 upon bacterial infiltration. By contrast, we could not identify genes of this type among the regulated transcripts in Gifu B-129 leaves.

Additional transcripts encoding for receptor-like kinases (RLK) were regulated in both ecotypes. Interestingly, as well as LRR-RLKs, it has been described that many RLK are also associated to signal transduction during pathogenesis [34]. In particular, some of these genes regulated in MG-20 and Gifu B-129 were referred by the functional map used in our analysis as RLK-4 homologues. Importantly, RLK-4 is induced by salicylic-acid treatment and pathogen infiltration in *Arabidopsis* [35]. Moreover, we also found three homologues to the *Arabidopsis* gene RLK-1 among the regulated transcripts in MG-20. RLK-1 was reported to function as a negative modulator of plant defense responses [36]. 

Host DAMPs are released upon microbial attack and recognized in *Arabidopsis* by a different kind of receptors, as PEPR1, PEPR2 and WAK kinases. These proteins were shown to be linked to a signaling cascade leading to increased expression of defense genes against pathogens [37,38]. Interestingly, a gene with homology to PEPR1 and four WAK-family transcripts were induced in MG-20. This observation correlates with the notorious tissue damage observed in this ecotype after bacterial infiltration. In turn, only one WAK-like gene was found to be regulated in Gifu B-129. 

Another kind of genes whose expression was induced by bacterial infiltration in both ecotypes is that of ionotropic glutamate receptors (iGRs). Interestingly, these proteins have been described recently to participate in pathogen recognition by mediating calcium signaling and nitric oxide (NO) production in a similar mechanism to that utilized by glutamate receptors in mammals [39]. 

Genes coding for plasma membrane-localized proteins known as non-race specific disease resistance1/harpin induced protein (NDR1/HIN1), play an important role functioning as central activators of defense signaling in *Arabidopsis* [40]. Upon infection with *Pto*, five NDR1/HIN1-like genes were up-regulated, and one down-regulated in MG-20, whereas two of them were induced in Gifu B-129. 

Our findings suggest that pathogen perception in *L. japonicus* involves similar components acting in this process in other species, and that the signaling might be regulated by the same families of receptors in both ecotypes. However, we found a higher proportion of regulated genes from this type in MG-20, suggesting that a more pronounced amplification of the response is required in this ecotype. By contrast, a less amplified response seems to be sufficient for successful defense in Gifu B-129.

### Stress related genes

Heat shock proteins (Hsps) play an important role in heat stress response in microorganisms, animals and plants [41]. Among Hsps, those belonging to the Hsp90 and Hsp70 family function as chaperones, and are known to be important in *Arabidopsis* for stomatal closure and physiological responses modulated by the plant hormone abscisic acid (ABA) [42]. Moreover, it has been demonstrated that the localization of these proteins, as well as their protein-binding capability are crucial for mounting plant defense responses [43–45]. This dependence of plant defense on Hsps might be exploited by microorganisms to cause a successful infection. Thus, plant Hsp70 is the major target of HopI1, a virulence effector of pathogenic *P. syringae* pv. *maculicola*. Importantly, this interaction suppresses plant defense and favors bacterial virulence [46]. 

Hsp expression seems to be important for triggering plant defense in MG-20, since transcriptomic data obtained in our work revealed a positive regulation of 7 Hsp90-like and 3 Hsp70-like transcripts in bacterial-challenged leaves of this ecotype. In turn, none of these genes were regulated in Gifu B-129 leaves under pathogenic conditions. 

### Redox homeostasis and detoxifying enzyme genes

ROS production is typically generated at the plant apoplast upon pathogen recognition and shows a biphasic pattern. Thus, a transitory increment in ROS concentration occurs within the first minutes after the contact with pathogens, which is followed by a period of sustained and perdurable ROS production [29]. Many reports have shown that this phenomenon not only leads to the activation of the defense responses associated to both PTI and ETI, but also causes direct injury to the pathogen. In addition, ROS also participate in the cross-linking of cell wall proteins so to constitute a physical barrier to the microorganism [47]. 

The enzyme NADPH oxidase (NOX), localized to the plasma membrane, is the main source of apoplastic ROS in response to pathogens. However, we didn’t find any NOX-like representative among the induced genes in MG-20 and Gifu B-129, suggesting that other sources of ROS may be in charge of the oxidative burst we evidenced in these plants. Many enzymes have been demonstrated to make a contribution to ROS accumulation in response to pathogens, like polyamine oxidases and cell wall peroxidases [29]. Interestingly, four putative peroxidases were up-regulated in both MG-20 and Gifu B-129 after *Pto* infiltration, even though we also found the up-regulation of other additional 16 genes in MG-20 and 1 in Gifu B-129. In turn, two putative polyamine oxidases were also induced by the pathogen in MG-20, whereas just one of these genes was induced in Gifu B-129. The participation of these alternative ROS-producing enzymes in the production of these reactive molecules during plant defense remains to be elucidated.

It is also known that plants down-regulate genes from the ROS-scavenging enzymatic battery to favor the accumulation of these reactive species [47]. Enzymatic members of this system are catalases (CAT), superoxide dismutases (SOD), ascorbate peroxidases (APX) and glutathione peroxidases (GPX). Our results revealed transcriptional down-regulation of several genes with homology to these enzymes upon bacterial infection. For instance, three SODs and five APXs were down-regulated in MG-20 during the pathogenic interaction. Equally, two of the APXs genes were also repressed in Gifu B-129 leaves. Strikingly, two members of the GPX family were induced in MG-20 in bacteria-infiltrated leaves, while the same genes were not regulated in Gifu B-129.

Cells also modulate the concentration of the antioxidant molecules ascorbate and glutathione to modify the redox capacity of the apoplast [47]. In this trend, it has been shown that in *Arabidopsis* neither of these molecules is accumulated within this compartment during *Pto* attack, but rather compartmentalized in vacuoles and peroxisomes, respectively. One of the main enzymes modulating the pool of apoplastic ascorbate is ascorbate oxidase (AO). High activities of this enzyme reduce antioxidant buffering and allow ROS to accumulate and persist [48]. In this trend, we found four genes with homology to AOs up-regulated in MG-20 leaves, and one in Gifu B-129. In turn, the level of glutathione in plants is modulated by the coordinated regulation of the enzymes involved in its synthesis and degradation [48]. Glutathione is produced via two successive enzymes: γ-glutamyl-cysteine synthase (γ-ECS) and glutathione synthetase (GS), whereas is degraded by γ- glutamyl transferase (γ-GT) and dipeptidase enzymes. Interestingly, our array data showed the up-regulation of one GS-like gene and the repression of one γ-GT-like gene in MG-20, whereas no regulation of these transcripts was detected in Gifu B-129. Thus, it seems that reduction of ascorbate concentration rather than that of glutathione may play an important role on plant defense in *L. japonicus* by favoring ROS accumulation.

At last, the enzyme glutathione S-transferase (GST) detoxifies a wide variety of toxic compounds, including by-products of ROS activity [49]. In concordance with the accumulation of these reactive molecules, we identified in our analysis a higher proportion of up-regulated GST-like transcripts in relation to those down-regulated (17:8 in MG-20 and 6:1 in Gifu B-129). 

Taken together, these results suggest that alternative ROS sources, other than NOX, are activated in both ecotypes of *L. japonicus* under pathogenic attack. This occurs with a simultaneous repression of detoxifying enzymes such as SOD and APX and reduction of the ascorbate concentration in order to favor ROS increment. However, glutathione concentration and GPX activity may be maintained high to avoid excessive ROS toxicity. By these means, both mechanisms could act coordinately to modulate ROS concentration as needed. 

### Transcription factors

The expression of a wide variety of plant transcription factors is modulated during plant biotic stress [50]. This process follows pathogen recognition and puts into operation a sophisticated network that regulates defense gene expression, where members of the same transcription factor family function as inducers and other members act as repressors.

Many transcription factors from the WRKY family are regulated in different plant species and appear to have a major role in plant immune responses. For instance, effectors like flg22 and chitin activate a MAPK cascade in *Arabidopsis* that up-regulates WRKY29 and WRKY22. Importantly, over-expression of WRKY29 or WRKY22 reduces susceptibility to pathogens in this species and rice, respectively [51,52]. A quite contrasting scenario is obtained if the expression of other WRKY proteins is adjusted. For instance, over-expression of WRKY4 enhances susceptibility to *Pto* in *Arabidopsis* [53]. Transcriptomic data obtained in our work identified 10 up-regulated and 2 down-regulated WRKY-like genes in MG-20 in response to bacterial attack. Four of the most regulated transcripts were also up-regulated in Gifu B-129. 

Another transcription factor family described to operate during pathogenic interactions is the basic-domain leucine-zipper (bZIP) group, in particular those of the subfamily TGA-bZIP [50]. In *Arabidopsis*, there are evidences demonstrating that many of these proteins interact with NPR1, a known mediator of the salicylic acid responses [54]. Our analysis in *L. japonicus* demonstrated that 2 TGA-bZIP-like genes were down-regulated in MG-20 following bacterial infection. Surprisingly, these genes show homology to the *Arabidopsis* TGA1 and TGA4 transcription factors, whose deletion has been shown to compromise the defense against *P. syringae* pv. *maculicola* [54]. In turn, a distinct TGA-bZIP factor was up-regulated in Gifu B-129 plants. This gene is weakly similar to *Arabidopsis* TGA10 and tobacco TGA2.1. However, neither TGA10 nor TGA2.1 seems to participate on plant defense [55,56]. Thus, it is possible that different legume-specific TGA proteins contribute to plant defense in *L. japonicus.*

ERFs (ethylene response factors) are also involved in pathogen-induced gene regulation and their transcription is activated following *Pto* infection [5]. Importantly, over-expression of these genes enhances plant resistance to pathogens in different plant species [57]. In this work, we found the induction of 9 ERF-like transcripts in MG-20, whereas 6 similar genes were repressed. In contrast, only four transcripts of this type were up-regulated in Gifu B-129 plants, while one was down-regulated. Notably, three of the induced ERFs in Gifu B-129 were among the most regulated transcription factors in MG-20. The closest homologues to these proteins were also induced in *Arabidopsis* after *P. syringae* infiltration, in particular against avirulent or non-host strains [58]. In addition, among the ERFs repressed in MG-20 we identified homologues to *Arabidopsis* ERF3 and ERF4. These two proteins were described to function as transcriptional repressor and are induced by incompatible pathogens [59]. In turn, an ERF5-like factor was also repressed in this ecotype, which has been described as positive regulator of plant defenses and confer resistance to pathogens when over-expressed [60]. Whether the repression of these genes affects normal plant defense responses in MG-20 will be explored in our future research. 

Myb and basic helix-loop-helix (bHLH) proteins constitute the largest families of transcription factors in *Arabidopsis* [61], and many of them are also induced by pathogen recognition and mediate hormone signaling [62]. In our analysis, we found 13 Myb-like genes up-regulated in MG-20, whereas 9 showed transcriptional repression during pathogenesis. In turn, just 4 up-regulated Myb proteins were present among the regulated genes in Gifu B-129. On the other hand, 15 bHLH genes in MG-20 were repressed during plant defense, a number consistently higher to those 5 up-regulated members of this family. Only two, up-regulated, bHLH-proteins were observed among the genes identified in Gifu B-129. Once again, as observed for ERFs and WRKYs, it should be noticed that all four Myb as well as the two bHLH proteins regulated in Gifu B-129 were among the transcripts of these families showing maximal regulation in MG-20. 

These results suggest that *L. japonicus* ecotypes modulate plant defense using similar components to those orchestrating this response in other plant species. Nevertheless, the role played by particular transcription factors should be evaluated. These proteins could show notorious functional differences with those described in non-legumes. 

### Hormone metabolism genes

Traditionally, it is considered that the molecular cascade activated by salicylic acid (SA) is essential for resistance against hemibiotrophic and biotrophic pathogens, and on the other hand, that jasmonic acid (JA) and ethylene signaling induce plant defense against nechrotrophic microorganisms [63]. These mechanisms are mostly antagonistic, however, it has also been demonstrated that synergistic regulation between them also exists, a feature important in order to fine-tune defense responses [64]. In addition, a good deal of research carried out over the last years has brought to light information supporting that other hormones, such as auxins and ABA also participate in plant defense activation [5]. The regulated genes identified in our work associated to plant hormone metabolism are discussed separately in the next paragraphs.

#### Salicylic acid

Plants synthesize SA by two different pathways using phenylalanine and chorismate as starting precursors [65]. The enzymes phenylalanine ammonia lyase (PAL) and isochorismate synthase (ICS) catalyze the first steps involved in phenylalanine and chorismate transformation, respectively. Importantly, the expression of PAL and ICS is induced under different biotic and abiotic stress conditions [65]. It is worthy to note at this point that cinnamate, the product of PAL activity, is a key intermediate of phenylpropanoid biosynthesis [66]. Therefore, as phenylpropanoids are also related to plant defense, we should take into account that PAL regulation would not merely modulate SA synthesis, but also that of a wider spectrum of defensive metabolites (genes related to phenylpropanoid metabolism will be discussed in a later subheading). Our results showed that *Pto* infection induced 4 and 1 PAL-like genes in MG-20 and Gifu B-129, respectively. In addition, one putative PAL gene was down-regulated in MG-20. However, we didn’t find the induction of any ICS-like gene in these ecotypes. In fact, one ICS-like gene was actually repressed in MG-20. These results are important since they suggest that the PAL-mediated pathway would be responsible for SA signaling activation in *L. japonicus*. Several genes responsible for SA conjugates production were also induced, which usually coincided with SA accumulation. In this trend, one UDP-glucose:SA glucosyltransferase (UGTase) and six *S*-Adenosyl-L-methionine:salicylic acid carboxyl methyltransferase (SAMT) were induced in MG-20, whereas one representative of each type was also up-regulated in Gifu B-129.

The activation of the SA pathway is associated to the production of several pathogenesis related (PR) proteins, defensive factors that contribute to resistance [67]. According to our transcriptomic results, 12 and 4 PR-like genes were found to be up-regulated after bacterial infiltration in MG-20 and Gifu B-129 plants, respectively. In *Arabidopsis*, the induction of the expression of PR genes mediated by SA pathway requires transcriptional co-regulators as NPR1 and Enhanced disease susceptibility 1 (EDS1) [67]. SA facilitates the movement of NPR1 into the nucleus, giving it the ability to interact with TGA-bZIP transcription factors to activate the expression of a plethora of defense genes, including PR1 [68]. It has also been documented the negative regulation of PR genes by NRP1-paralogues as NPR3 and NPR4 [69]. On other hand, EDS1 shows homology to lipases and has been demonstrated to collaborate with NB-LRR genes to trigger resistance against biotrophic microorganisms [70]. Different EDS-like proteins, as EDS5 and SID2 are also indispensable for a full expression of PRs and it has been reported that knock-down mutants in these genes shows higher susceptibility to pathogens [71]. In our analysis, one transcript with homology to NPR3 and one EDS5-like were found negatively regulated in MG-20 as result of the infection. None of these genes were regulated in Gifu B-129.

As a whole, these results suggest that induction of the SA pathway occur in *L. japonicus* under the attack of *Pto*. However, given the complexity of the regulation of the defense mechanisms, we envision that other defense-related hormones might also take a substantial role during these responses.

#### Jasmonic acid

JA is a lipid-derived hormone whose synthesis depends on the successive activities of the enzymes lipoxygenase (LOX), allene oxide synthase (AOS), allene oxide cyclase (AOC) and 12-oxophytodienoate-10,11-reductase 3 (OPR3), followed with three steps of side-chain shortening β-oxidation [72]. Moreover, the biosynthetic enzyme jasmonoyl-isoleucine synthetase (JAR1) forms the JA-isoleucine conjugate (JA-Ile), one of the most active natural jasmonate derivatives. The bioactive end-product of the hormone is a ternary complex comprising JA-Ile, the F-box protein coronatine insensitive 1 (COI1) and one of several JAZ proteins [72]. 

Our analysis suggests the activation of JA synthesis in *L. japonicus* MG-20 upon bacterial challenge. This is supported by the induction of 4 LOX, 3 AOS, 1 AOC and 2 OPR3 transcripts, while only one LOX and one OPR3 were down-regulated under the same conditions. By contrast, a quite different scenario was observed in Gifu B-129 leaves, where biosynthesis of JA did not seem to be activated. Thus, only one OPR3 transcript was induced, whereas one LOX was down-regulated. In addition, the activation of JA signaling in MG-20 may not be dependent on classical mediators, since two JAR1 and one COI1 homologues were down-regulated under pathogenic conditions. The existence of a COI1-independent JA pathway has been described in *Arabidopsis* and tomato [73,74]. 

Interestingly, we also identified two pathogen-inducible α-dioxygenases among the up-regulated genes in MG-20. This kind of enzymes catalyzed the alternative oxidation of unsaturated lipids to generate 2-hydroperoxy fatty acids. Many genes of this kind are induced in other plant species after inoculation with pathogens and have been described to contribute to plant defense [75].

#### Ethylene

Ethylene often works synergistically with JA during pathogen attack [63]. It is synthesized from S-adenosyl-methionine by the action of two key enzymes, 1-aminocyclopropane-1-carboxylic acid synthase (ACS) and 1-aminocyclopropane-1-carboxylic acid oxidase (ACO). Upon bacterial infiltration, 3 ACS and 5 ACO genes were up-regulated in *L. japonicus* MG-20 leaves, whereas just one member of the first group was induced in Gifu B-129. These results suggest that the ethylene response, as well as that of JA, might be active in MG-20 but has a minor role in Gifu B-129. 

#### ABA

ABA plays a crucial role in the regulation of plant growth and adaptation to abiotic stress [76]. The participation of this hormone in the response against invading microorganisms seems to be quite complex and depends on the type of the pathogenic interaction [77], but it has been generally associated to the negative regulation of defense mechanisms. In this trend, some authors have suggested that *Pto* induces ABA biosynthesis in *Arabidopsis* as a virulence strategy [78]. ABA is synthesized by plants through the epoxidation of the carotenoids zeaxanthin and antheraxanthin, catalyzed by a zeaxanthin epoxidase (ZEP). This reaction is followed by the actions of the enzymes 9-cis-epoxycarotenoid dioxygenase (NCED), short-chain alcohol dehydrogenase/reductase (SDR), and aldehyde oxidase (AAO) [79]. In MG-20, most of these genes from the ABA metabolism were negatively regulated after *Pto* infiltration. Thus, we found four ZEPs, one NCED, one SDR and two AAO down-regulated under this treatment, suggesting that ABA biosynthesis is blocked. This feature indicates that *Pto* is unable to manipulate ABA metabolism in *L. japonicus* as it does in other plant hosts. In addition, as ABA plays a crucial role during drought stress, down-regulation of this pathway in MG-20 is congruent with the observation of mild or partial dehydratation in the area surrounding the infiltration point. No representatives from these categories were found among the pathogen-regulated genes in Gifu B-129. 

#### Auxins

Indole-3-acetic acid (IAA) is the most abundant naturally occurring auxin in plants. Some emerging evidence demonstrated that this hormone enhances plant disease susceptibility, and that down-regulation of IAA signaling is part of the defense responses occurring in plants [80]. This effect seems to be explained by the IAA-mediated induction of expansin genes (thence loosening the cell wall) and suppression of the SA signaling pathway. In addition, defense modulation by SA and JA-independent mechanisms were also described for this hormone [81,82]. The biosynthesis of IAA is quite complex and involves many pathways that may be grouped into tryptophane (Trp)-dependent and Trp-independent mechanisms. These metabolic routes, along with IAA conjugation, are the main mechanisms utilized by plants to regulate this hormone activity. The action of auxins is mediated by the induction of three gene families, the transcriptional repressors Aux/IAA, GH3 and small auxin-up RNA (SAUR) genes [77]. GH3 genes encode IAA-amino synthetases and have also been implicated in the mounting of plant defense responses. Thus, the over expression of the GH3.8 protein in rice prevents free IAA accumulation and lead to enhanced disease resistance to *Xanthomonas oryzae* *pv. oryzae* [81]. Similarly, GH3.5 modulates both the SA as well as the IAA signaling during biotic stress in *Arabidopsis* [83]. 

No transcripts related with the IAA biosynthetic pathway were regulated in *L. japonicus* after inoculation with *Pto*. However, our microarray results showed that 4 and 2 GH3-likes transcripts were up-regulated in MG-20 and Gifu B-129, respectively. We also detected several transcripts with homology to IAA-amino acid hydrolases in MG-20, but not in Gifu B-129, one of them being induced and three down-regulated. These genes are shown to release free IAA from IAA-amino acid conjugates [84]. Altogether, these data seems to indicate that free IAA concentration is diminished as a response to bacterial attack in *L. japonicus*.

### Phenylpropanoid metabolism

Phenylpropanoids are a variety of compounds derived from phenylalanine with very important roles in plant defense and survival [66]. The first steps in the phenylpropanoid pathway comprehend the reactions catalyzed by PAL (already analyzed in a previous subheading in relation to the SA pathway), cinnamate 4-hydroxylase (C4H) and 4-coumarate:CoA ligase (4CL). The end product of these enzymes is *p*-coumaroyl-CoA, the intermediate compound used by different metabolic routes to produce phenylpropanoid derivates as lignins, phytoalexins, and flavonoids (flavones, isoflavonoids and anthocyanins/proanthocyanidins) [66]. Besides the PAL genes regulated as described before, we also found the induction of two C4H genes in both ecotypes, indicating that the phenylpropanoid metabolism is induced in response to *Pto*.

The activity of chalcone synthase (CHS) is the first step involved in flavonoid biosynthesis, diverting the pathway away from lignins. This enzyme is induced in plants under pathogenic conditions and is important for resistance against bacteria and fungi. Accordingly, we found 6 CHS-like transcripts whose expression was induced in MG-20 by *Pto*. In turn, 2 of these transcripts were similarly induced in Gifu B-129. These results suggest that flavonoid biosynthesis is activated similarly in both ecotypes upon bacterial attack. In agreement with this, many reductase enzymes important in flavonoid metabolism (dihydroflavone reductase, DFR; isoflavone reductase, IFR; vestitone reductase, VR) were also activated. IFR and VR participate in the formation of phytoalexins, low molecular weight flavonoids showing antimicrobial activity and synthesized de novo in response to pathogens [85]. In the genus *Lotus*, the main phytoalexin biosynthesized is vestitol, which belongs to the class of isoflavone aglycone [86]. DFR is an enzyme also involved in anthocyanin and proanthocyanidin metabolism, another kind of flavonoid-derived compounds. The branch-point enzyme in the flavonoid pathway that directs the synthesis toward anthocyanins and proanthocyanidins is flavanone 3-hydroxylase (F3H). In our data, we found five F3H-like genes repressed in MG-20, whereas none of this kind were found in Gifu B-129. Thus, we conclude that flavonoid metabolism in *L. japonicus* prioritizes phytoalexin production when plants are confronted to *Pto*. A similar conclusion was drawn when the phenylpropanoid metabolism was evaluated in *G. max* plants under the attack of *P. syringae* pv. *glycinea* [87]. 

Lignification is a well known mechanism of plant cell wall reinforcement to occur in many plant-pathogen interactions [88]. Several lignin biosynthetic genes were positively regulated in MG-20, including cinnamyl alcohol dehydrogenases (CAD), cinnamyl-CoA reductases (CCR), and caffeic acid O-methyltransferases (COMT). Thus, these results suggest that lignin metabolism may play an important role in the defense of this ecotype. In turn, just one COMT gene was shown to be positively regulated in Gifu B-129. 

### Verification of array data by qRT-PCR

**Figure 5:**
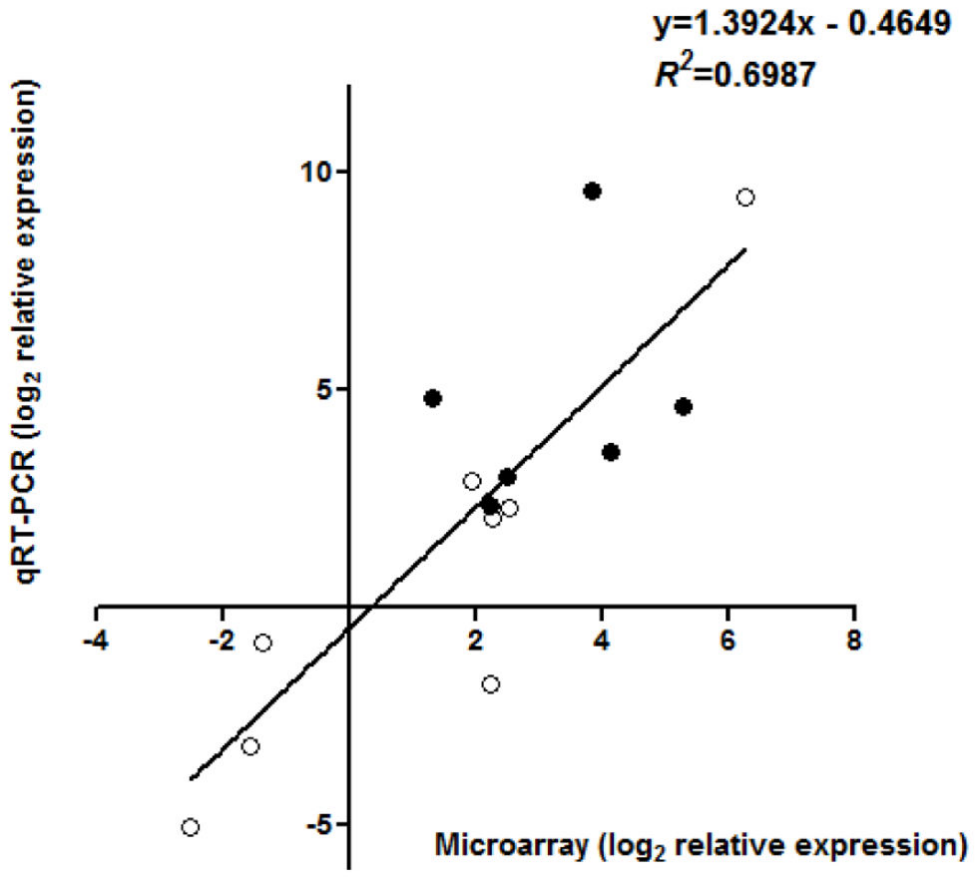
Comparison of microarray and quantitative real-time PCR data for selected genes. Symbols represent mean expression levels in bacterial-infiltrated relative to mock-inoculated leaflets of Gifu B-129 (●) or MG-20 (○).

Using total RNA from independent biological replicates we validated the microarray expression data of 14 selected genes by qRT-PCR. These genes, representing different biological functions, were selected on the basis of our microarray results because they were induced in either MG-20 and/or Gifu B-129 following bacterial infiltration. Linear regression analysis of microarray and qRT-PCR values yielded a value of R^2^=0.6987 (Pearson’s correlation r value of 0.8359) and a slope of 1.3924 (Figure 5), demonstrating that although the extent of transcription regulation varied between both techniques, the patterns observed were similar (in fact, the regulation of 13 out of the 14 genes was confirmed). Thus, we inferred that our microarray analysis accurately identified regulated genes due to bacterial inoculation. See table S3 for a further description of the genes evaluated in this experiment. 

### Comparison between mock-inoculated leaves

**Figure 6:**
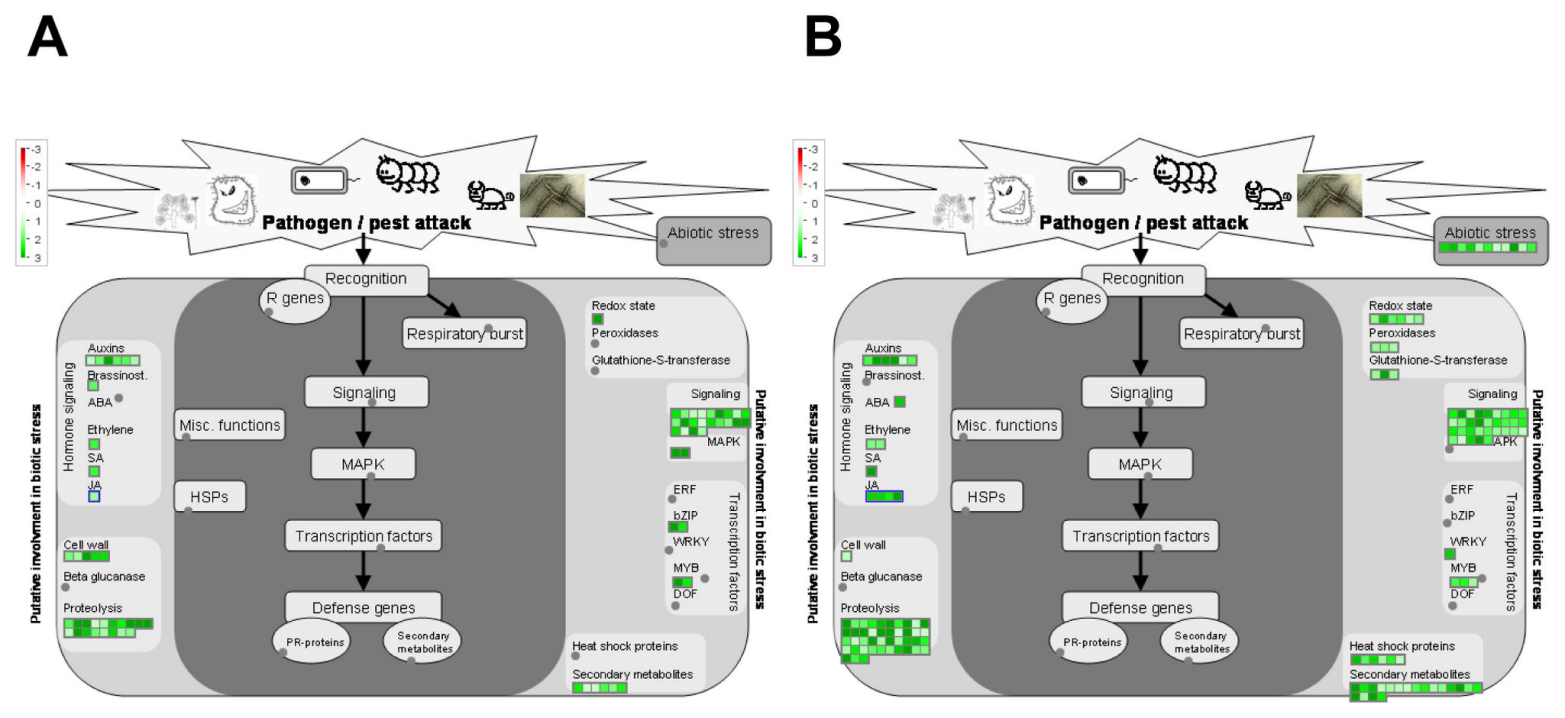
MapMan illustration depicting biotic stress-related transcripts with a relative higher expression when mock-inoculated samples are compared. Transcriptomic data from mock-inoculated leaves of MG-20 and Gifu B-129 were compared to each other. Genes that were shown to be differentially expressed in MG-20 (A) and Gifu B-129 (B) were mapped using the MapMan software. Picture shows only those genes related to biotic stress. Log fold change ratios are indicated as a gradient of green.

A preliminary comparison of the microarray expression data between mock-inoculated samples from both ecotypes revealed that many genes were significantly altered in abundance. We selected 6 of these genes and confirmed the expression ratio by qRT-PCR but this time using non-infiltrated samples (Table 2), indicating that the infiltration procedure does not cause a major perturbation on plant gene expression. This observation provided us the opportunity to compare the transcriptomic data between mock-inoculated samples so to evaluate the differences in basal gene expression that could explain the higher tolerance of Gifu B-129. Interestingly, our analysis demonstrated that the expression of 880 transcripts was significantly different between MG-20 and Gifu B-129. A total of 195 of these genes were mapped as biotic stress-related genes (Figure 6). Remarkably, the majority of these genes, exactly 121, showed a higher expression in Gifu B-129, whereas 74 were highly expressed in MG-20. The most notable differences between both ecotypes were found in categories as secondary metabolism, Hsps, redox state modulators and abiotic stress, all of them being more represented in Gifu B-129. 

**Table 2:** Comparison of the relative expression levels in Gifu B-129 vs. MG-20 of selected genes as determined by microarray (mock-inoculated samples) and qRT-PCR (non-inoculated samples).

**Lotus Affymetrix ID**	**Gene description**	**Microarray data (log_2_ mean relative expression)**	**p value for microarray **	**qRT-PCR data (log_2_ mean relative expression)**	**p value for qRT-PCR**
ljwgs_023901.2_at	Flavonol synthase	-2.345	0.000795	-0.737	0.0282
ljwgs_025651.1.1_at	Disease resistance-responsive family protein	4.800	0.000010	1.778	0.037
chr5.tm1493.8_at	GID1-like gibberellin receptor	3.448	0.004931	2.345	0.022
chr3.cm0216.2_at	IFR-like protein	2.508	0.000745	2.954	0.009
chr5.cm0344.11_at	Somatic embryogenesis receptor kinase	7.398	0.000005	8.117	0.010
chr5.cm0062.23_at	Hsp DnaJ	2.661	0.000683	8.211	0.016

Among the genes more expressed in Gifu B-129 we found a group that did not respond to bacterial infiltration in any case, genes that were not regulated in Gifu B-129 but did so in MG-20 after treatment, and last, genes that respond to the bacteria in both ecotypes. Genes from the first group we identified three HSPs and other stress related proteins, as UV-damaged DNA binding factors, universal stress proteins, GSTs, LOXs and 4CL (Table S1, last column). The second group was composed of a SERK-like kinase, a PEPR1 homologue, an IGr receptor, peroxidases, a WRKY type transcription factor, a pathogen-inducible alpha dioxygenase 1, and several phenylpropanoid metabolic genes as DFR, VR and COMT. Finally, the third group was made up of an NDR1/HIN1 homologue, a peroxidase, a GST, and a DFR gene.

We hypothesize that a higher expression of some of these genes could improve the fitness of Gifu B-129 against the bacteria by restricting its growth within the leaves or even speeding up the activation of the defense responses. If this is the case, this ecotype could detect the pathogen earlier and induce a faster response to cope with it, offering an explanation for the better performance of this ecotype under *Pto* attack. Further research should be undertaken to unravel the participation of these genes on plant defense.

## Conclusions

Although many studies were carried out in other legume species, the interaction between bacterial pathogens and *L. japonicus* has received very little attention. Perhaps the relatively recent emergence of this plant as a model species, in addition to the lack of a robust pathosystem (which does not occur in other legume models such as *M. truncatula* and *G. max*), are among the main factors associated with the absence of research on this species. Therefore, the need to characterize and explore the responses that occurs in *L. japonicus* against pathogenic microorganisms is unquestionable. The development and adoption of a model pathosystem in this legume will certainly potentiate studies on the field, complementing the research carried out in other members of the family and, by the same token, providing us with knowledge that can be extrapolated to crop breeding. With this premise as our main aim, in the present work we evaluated the behavior of two phenotypically contrasting accessions of *L. japonicus* during the attack of the bacteria *Pto*. Interestingly, these accessions showed a quite different tolerance against the pathogen, suggesting that natural variation occurs in the defense mechanisms used by distinct genotypes of this plant species. 

No symptoms were visible when the ecotype Gifu B-129 was challenged to the bacteria. Our further analysis demonstrated that the microorganism was still able to survive within the leaves during the course of the experiment, even though the growth rate was very slow. Interestingly, very early after bacterial infiltration, a strong ROS production was observed in leaves of this ecotype. By contrast, when the ecotype MG-20 was confronted to the pathogen, light chlorotic spots appeared and quickly progressed covering larger areas to conclude in tissue collapse after 48-72 h. In this case, bacterial count consistently increases more than three-fold (log_10_ scale) during the course of the experiment. Moreover, bacterial infiltration also elicited ROS accumulation in MG-20, even though the intensity of the response was lower and appeared later. Importantly, these effects were limited to the infiltrated leaflet, since no symptoms or bacterial invasion could be determined in adjacent leaflets of the same leaf. 

We detected several genes being regulated in these ecotypes in response to the bacteria. The degree of regulation was higher in MG-20, where the expression of 5217 transcripts significantly changed after bacterial infiltration. Gifu B-129 transcriptome was far less affected, since 458 passed our filter criteria. Most of the genes regulated in Gifu B-129 were similarly regulated in MG-20, providing us a set of genes commonly regulated in this legume species as a response to the pathogen. Two considerations must be taken into account at this point when the lists of regulated genes are analyzed. First, it should be kept in mind that some of the genes regulated in MG-20 may be responding to the tissue damage produced by bacterial multiplication, even though at the time the samples were taken only mild symptoms were evidenced in this ecotype. Another point to be considered is that microarray data were obtained at only one time point following bacterial infiltration (24 hpi). This means that earlier gene expression fluctuations could have been missed under our experimental setup. 

Functional classification of regulated genes identified several transcripts involved in pathogen perception and amplification of the defense response. This was also accompanied by the induction of ROS-producing enzymes and repression of the scavenging battery, which should be responsible for ROS accumulation in infected tissues. Our data also suggest that SA signaling mediates the activation of the defense responses, leading to the expression of several PR genes. However, MG-20 plants also showed a substantial activation of the JA/Ethylene pathway, which might be advantageous for the pathogen and could explain in part the higher rate of multiplication of *Pto* in this ecotype.

Importantly, many genes related to plant defense showed a higher basal expression in Gifu B-129, particularly those involved in lignine biosynthesis, ROS homeostasis and general stress defense. The higher expression of these genes could prepare the plant to readily deal with the bacteria immediately or even before its recognition, an effect that could be translated in the appearance of fewer symptoms. 

Our future work will look at the responses occurring in *L. japonicus* during the attack of other legume-infecting pathovars of *P. syringae*. This will give us a valuable set of data that may help to identify the main mechanisms deployed by this legume to avoid the disease. In addition, we foresee that this information could be compared to that obtained in past studies on the interaction between *L. japonicus* and beneficial bacterial symbionts and mycorrhizal fungi, revealing interesting aspects of plant gene expression during the contact with a wide variety of microorganisms.

## Supporting Information

Table S1 **List of genes differentially regulated in MG-20 and Gifu B-129 under pathogenic conditions and those differentially expressed in mock-inoculated Gifu B-129 relative to mock-inoculated MG-20 .**
(XLS)

Table S2 **Selected genes with differential expression in L. japonicus ecotypes MG-20 and Gifu B-129.**
(DOC)

Table S3 **Microarray and qRT-PCR expression values for genes used to build Figure 5.**
(DOC)
