# Supplementary material for: Defense Responses in Two Ecotypes of Lotus japonicus against Non-Pathogenic Pseudomonas syringae
Source: PLoS One. 2013 Dec 11;8(12):e83199. doi: 10.1371/journal.pone.0083199 (PMC3859661; doi:10.1371/journal.pone.0083199)
Supplement: Table S2 — Selected genes with differential expression in L. japonicus ecotypes MG-20 and Gifu B-129. (DOC) [file pone.0083199.s002.doc]

**Table S2. Selected genes with differential expression in leaves of *L. japonicus* ecotypes MG-20 and Gifu B-129.** Values are log_2_ mean relative expression in bacterial-infiltrated MG-20 leaves (third column), bacterial-infiltrated Gifu B-129 leaves (fourth column), and the comparison between mock-inoculated Gifu B-129 vs. MG-20 leaves (fifth column).

| **Lotus Affymetrix ID** | **Gene annotation** | **Log_2_ mean relative expression** | | |
| --- | --- | --- | --- | --- |
|  |  | **MG-20** | **Gifu** | **Gifu/MG-20** |
| **Pathogen perception and defense signaling** |  |  |  |  |
| chr6.cm0314.39_at | SERK-like kinase | 1.409 | n.r. |  |
| chr5.cm0200.92.1_at | SERK-like kinase | 1.611 | n.r. |  |
| chr5.cm0344.11_at | SERK-like kinase | 3.267 | n.r. | 7.398 |
| TM0855.10.1_at | EFR protein | 2.922 | n.r. |  |
| ljwgs_051950.1_s_at | RLK-4-like kinase | -1.789 | n.r. |  |
| chr1.cm0088.82_at | RLK-4-like kinase | -1.569 | n.r. |  |
| chr3.cm0005.60_at | RLK-4-like kinase | 2.571 | n.r. |  |
| chr4.tm1415.15.1_at | RLK-4-like kinase | 2.711 | n.r. |  |
| ljwgs_056302.1_at | RLK-4-like kinase | 3.882 | 2.569 |  |
| ljwgs_058803.1_at | RLK-1-like kinase | -2.591 | n.r. |  |
| chr5.cm0095.36_s_at | RLK-1-like kinase | -1.415 | n.r. |  |
| ljwgs_015301.2_at | RLK-1-like kinase | 1.748 | n.r. |  |
| ljwgs_146892.1_at | PEPR1-like | 3.167 | n.r. | 2.089 |
| ljwgs_019770.1_at | WAK-family | 1.592 | n.r. |  |
| chr1.cm0064.22_at | WAK-family | 2.089 | n.r. |  |
| chr1.tm1124.12_at | WAK-family | 2.095 | n.r. |  |
| ljwgs_086310.1_at | WAK-family | 2.479 | n.r. |  |
| ljwgs_027326.1_at | WAK-family | 5.140 | 2.502 |  |
| chr1.CM0133.4_s_at | IGr | 1.674 | n.r. |  |
| chr1.CM0133.24_s_at | IGr | 2.141 | n.r. | 2.417 |
| chr1.CM0133.17_at | IGr | 2.312 | n.r. |  |
| ljwgs_014308.2_at | IGr | 4.794 | 3.197 |  |
| chr1.CM0133.21_at | IGr | n.r. | 1.264 |  |
| ljwgs_017693.1_at | NDR1/HIN1-like | -1.814 | n.r. |  |
| chr1.CM0088.86_at | NDR1/HIN1-like | 1.069 | n.r. |  |
| chr1.CM0980.20_at | NDR1/HIN1-like | 3.744 | n.r. |  |
| ljwgs_014095.2_at | NDR1/HIN1-like | 4.173 | n.r. |  |
| ljwgs_073332.1_at | NDR1/HIN1-like | 4.922 | 2.584 | 3.132 |
| chr1.CM0980.23_at | NDR1/HIN1-like | 5.581 | 2.92 |  |
| **Heat shock proteins** |  |  |  |  |
| chr2.CM0210.7_at | HSP90 | 1.039 | n.r. |  |
| CM1324.27.1_at | HSP90 | 1.069 | n.r. |  |
| chr3.CM0253.33.1_at | HSP90 | 1.179 | n.r. |  |
| chr3.CM0253.33.2_at | HSP90 | 1.371 | n.r. |  |
| chr2.CM0191.25.1_at | HSP90 | 1.676 | n.r. |  |
| ljwgs_062422.1_s_at | HSP90 | 1.934 | n.r. |  |
| ljwgs_033122.2_at | HSP90 | 1.943 | n.r. |  |
| chr3.CM0452.21_at | HSP70 | 1.254 | n.r. |  |
| chr6.CM0055.1_at | HSP70 | 1.286 | n.r. |  |
| ljwgs_034228.1_at | HSP70 | 3.022 | n.r. |  |
| ljwgs_014093.1_at | HSP70 | n.r. | n.r. | 1.990 |
| ljwgs_062145.1_at | HSP70 | n.r. | n.r. | 2.245 |
| ljwgs_147202.1_at | HSP70 | n.r. | n.r. | 2.815 |
| **Redox homeostasis** |  |  |  |  |
| chr5.cm0158.40_at | Peroxidase-like | 1.364 | n.r. |  |
| ljwgs_027021.1_at | Peroxidase-like | 1.397 | n.r. |  |
| TM0879.19_s_at | Peroxidase-like | 1.814 | n.r. |  |
| ljwgs_002758.2_at | Peroxidase-like | 1.925 | n.r. |  |
| TM0879.22_at | Peroxidase-like | 2.085 | n.r. | 1.556 |
| chr5.cm0052.9.1_at | Peroxidase-like | 2.127 | n.r. |  |
| chr5.cm0052.5_at | Peroxidase-like | 2.183 | 1.805 |  |
| chr5.cm0052.5.1_at | Peroxidase-like | 2.284 | n.r. |  |
| ljwgs_016682.1_at | Peroxidase-like | 2.366 | n.r. |  |
| ljwgs_043353.1_at | Peroxidase-like | 2.618 | n.r. |  |
| ljwgs_018216.1_at | Peroxidase-like | 3.065 | 4.319 |  |
| ljwgs_016747.1_at | Peroxidase-like | 3.258 | n.r. |  |
| chr3.cm0135.12.1_at | Peroxidase-like | 3.717 | n.r. |  |
| chr1.cm0248.26_at | Peroxidase-like | 3.750 | n.r. | 1.670 |
| ljwgs_103604.1_at | Peroxidase-like | 3.899 | 1.846 |  |
| ljwgs_026466.1_at | Peroxidase-like | 3.971 | n.r. |  |
| chr3.cm0135.19_at | Peroxidase-like | 4.003 | n.r. |  |
| TM1644.19_at | Peroxidase-like | 4.083 | 2.149 | 1.404 |
| ljwgs_010765.2_at | Peroxidase-like | 4.136 | n.r. |  |
| chr3.cm0135.12_at | Peroxidase-like | 4.752 | n.r. |  |
| ljwgs_064507.1_at | Peroxidase-like | n.r. | 3.375 |  |
| ljwgs_030331.1.1_at | Polyamine oxidase | 1.124 | 1.437 |  |
| ljwgs_077893.1_s_at | Polyamine oxidase | 1.286 | n.r. |  |
| chr3.CM0396.35_at | Superoxide dismutase | -2.161 | n.r. |  |
| ljwgs_130109.1_s_at | Superoxide dismutase | -1.767 | n.r. |  |
| ljwgs_032199.1_at | Superoxide dismutase | -1.197 | n.r. |  |
| ljwgs_030740.1_s_at | Ascorbate peroxidase | -3.207 | n.r. |  |
| ljwgs_139156.1_s_at | Ascorbate peroxidase | -2.71 | -2.268 |  |
| chr1.CM0016.20_at | Ascorbate peroxidase | -2.430 | -2.057 |  |
| TM0874.9_at | Ascorbate peroxidase | -1.128 | n.r. |  |
| ljwgs_054875.1_at | Ascorbate peroxidase | -1.113 | n.r. |  |
| chr4.CM0042.46_at | Glutathione peroxidase | 2.139 | n.r. | 1.646 |
| ljwgs_038927.1_s_at | Glutathione peroxidase | 2.093 | n.r. | 1.212 |
| ljwgs_025437.1.1_at | Ascorbate oxidase | 1.072 | n.r. |  |
| ljwgs_036352.1_at | Ascorbate oxidase | 2.018 | n.r. |  |
| chr3.cm0208.34_at | Ascorbate oxidase | 4.221 | n.r. |  |
| chr2.CM0002.36_at | Ascorbate oxidase | 4.954 | 3.569 |  |
| gi37222543_at | Glutathione synthetase | 2.382 | n.r. |  |
| ljwgs_027953.1_at | γ-glutamyl-transferase | -1.385 | n.r. |  |
| **Detoxification enzymes** |  |  |  |  |
| chr6.CM0066.38.3_at | GST | -3.470 | n.r. |  |
| ljwgs_048157.1_s_at | GST | -1.994 | n.r. |  |
| ljwgs_031763.1_s_at | GST | -1.867 | n.r. |  |
| ljwgs_042082.1_at | GST | -1.754 | n.r. |  |
| ljwgs_128714.1_s_at | GST | -1.466 | n.r. |  |
| ljwgs_037557.1_at | GST | -1.306 | -1.150 |  |
| chr4.CM0558.29_s_at | GST | -1.208 | n.r. |  |
| chr6.CM0836.4.1_x_at | GST | -1.073 | n.r. |  |
| ljwgs_031569.1_at | GST | 1.150 | n.r. |  |
| ljwgs_072403.1_s_at | GST | 1.552 | n.r. |  |
| ljwgs_014259.1_at | GST | 1.559 | n.r. |  |
| chr4.CM0046.39_at | GST | 1.736 | n.r. |  |
| ljwgs_055009.1_at | GST | 1.905 | 1.724 |  |
| chr5.CM0909.43_at | GST | 1.953 | n.r. |  |
| ljwgs_018999.1_at | GST | 2.073 | n.r. |  |
| ljwgs_020173.1_s_at | GST | 2.092 | n.r. |  |
| chr4.CM0046.42_at | GST | 2.374 | 2.122 | 1.546 |
| chr1.TM0240.20_at | GST | 2.638 | n.r. |  |
| chr5.CM0909.46_at | GST | 3.386 | n.r. |  |
| chr5.CM0909.51_at | GST | 3.768 | n.r. |  |
| chr5.CM0909.52_at | GST | 4.497 | n.r. |  |
| chr5.CM0909.50_at | GST | 6.135 | 3.853 |  |
| chr5.CM0909.59_at | GST | 6.240 | 4.294 |  |
| chr5.CM0909.45_at | GST | 6.839 | 4.074 |  |
| ljwgs_061228.1_at | GST | 7.452 | 4.614 |  |
| ljwgs_028218.2_s_at | GST | n.r. | n.r. | 1.564 |
| ljwgs_040903.1_at | GST | n.r. | n.r. | 5.835 |
| **Transcription factors** |  |  |  |  |
| ljwgs_055792.1_at | WRKY | -2.555 | n.r. |  |
| ljwgs_058052.1_at | WRKY | -1.405 | n.r. |  |
| ljwgs_050580.1_at | WRKY | 1.376 | n.r. |  |
| ljwgs_050544.1_at | WRKY | 1.647 | n.r. |  |
| chr5.CM0239.70_at | WRKY | 1.901 | n.r. |  |
| TM0801.7_at | WRKY | 3.061 | n.r. |  |
| chr3.CM0243.7_at | WRKY | 3.196 | n.r. |  |
| chr4.CM0333.3_at | WRKY | 3.223 | 2.364 |  |
| ljwgs_008140.3_at | WRKY | 3.579 | 1.949 |  |
| chr5.CM0040.86_at | WRKY | 3.629 | n.r. | 2.692 |
| chr1.CM0123.35.1_at | WRKY | 4.480 | 3.113 |  |
| TM0742.9_at | WRKY | 5.729 | 3.772 |  |
| chr5.CM0909.32_at | TGA-bZIP | -2.133 | n.r. |  |
| TM1262.25_at | TGA-bZIP | -1.539 | n.r. |  |
| ljwgs_061086.1.1_at | TGA-bZIP | n.r. | 2.489 |  |
| ljwgs_141699.1_at | ERF | -2.726 | n.r. |  |
| ljwgs_019003.1_at | ERF | -2.676 | n.r. |  |
| CM0905.10_at | ERF | -1.962 | n.r. |  |
| ljwgs_004289.2_at | ERF | -1.333 | n.r. |  |
| ljwgs_026196.1_at | ERF | -1.145 | n.r. |  |
| chr2.TM1285.7.1_at | ERF | -1.133 | n.r. |  |
| chr3.CM0080.34_s_at | ERF | 1.953 | n.r. |  |
| ljwgs_025266.1_at | ERF | 2.036 | n.r. |  |
| ljwgs_091106.1_at | ERF | 2.137 | n.r. |  |
| chr5.CM0052.19_at | ERF | 3.409 | n.r. |  |
| ljwgs_028626.1_at | ERF | 4.425 | 5.374 |  |
| chr1.CM0094.29_at | ERF | 4.828 | 2.131 |  |
| chr1.CM0032.5_at | ERF | 6.319 | n.r. |  |
| chr2.TM1756.16_at | ERF | 6.516 | 3.301 |  |
| ljwgs_100509.2_at | ERF | 5.273 | 3.669 |  |
| ljwgs_134387.1_at | ERF | n.r. | -1.533 |  |
| chr4.BM1698.6_at | Myb | -2.699 | n.r. |  |
| ljwgs_015858.1_at | Myb | -1.939 | n.r. |  |
| chr5.TM0562.28_at | Myb | -1.820 | n.r. |  |
| TM0874.11_at | Myb | -1.699 | n.r. |  |
| ljwgs_025851.1_at | Myb | -1.536 | n.r. |  |
| gi60476407_at | Myb | -1.408 | n.r. |  |
| chr3.TM0022.22_s_at | Myb | -1.291 | n.r. |  |
| ljwgs_072779.1_at | Myb | -1.273 | n.r. |  |
| ljwgs_079435.2_at | Myb | -1.231 | n.r. |  |
| ljwgs_057664.1_at | Myb | 1.807 | n.r. |  |
| ljwgs_014958.1_at | Myb | 1.981 | n.r. |  |
| ljwgs_122488.1_s_at | Myb | 2.097 | n.r. |  |
| ljwgs_070863.1_at | Myb | 2.204 | n.r. |  |
| ljwgs_007368.1_at | Myb | 2.851 | n.r. |  |
| chr2.TM0652.9_at | Myb | 2.962 | n.r. |  |
| chr1.CM0600.9_at | Myb | 3.449 | n.r. |  |
| ljwgs_057552.1_at | Myb | 5.086 | n.r. |  |
| chr1.TM1643.4.1_s_at | Myb | 5.611 | 3.542 |  |
| chr3.CM0711.17_at | Myb | 5.719 | n.r. |  |
| chr1.TM1643.3_at | Myb | 5.868 | 3.428 |  |
| chr4.CM0073.86_at | Myb | 5.914 | 3.501 |  |
| ljwgs_025887.1_at | Myb | 6.608 | 3.808 |  |
| ljwgs_054486.1_at | bHLH | -2.046 | n.r. |  |
| ljwgs_006701.1_at | bHLH | -2.903 | n.r. |  |
| chr5.cm0095.14_at | bHLH | -2.947 | n.r. |  |
| ljwgs_042411.1_at | bHLH | -2.412 | n.r. |  |
| chr6.TM1763.7_at | bHLH | -2.384 | n.r. |  |
| chr2.CM0002.22_at | bHLH | -2.361 | n.r. |  |
| ljwgs_053677.1.1_at | bHLH | -2.100 | n.r. |  |
| ljwgs_050347.1_at | bHLH | -2.040 | n.r. |  |
| ljwgs_066028.1_at | bHLH | -1.922 | n.r. |  |
| ljwgs_030731.1_at | bHLH | -1.901 | n.r. |  |
| ljwgs_059025.1_at | bHLH | -1.751 | n.r. |  |
| chr1.CM0318.9_at | bHLH | -1.578 | n.r. |  |
| chr2.CM0177.91_at | bHLH | -1.448 | n.r. |  |
| chr5.CM0048.61_at | bHLH | -1.070 | n.r. |  |
| chr2.CM0124.32.1_at | bHLH | -1.025 | n.r. |  |
| TM0914.6_at | bHLH | 1.228 | n.r. |  |
| chr1.CM0393.64_at | bHLH | 1.643 | n.r. |  |
| TM1571.9_at | bHLH | 3.335 | n.r. |  |
| chr5.TM0186.14_at | bHLH | 3.772 | 3.097 |  |
| ljwgs_025587.1_at | bHLH | 6.204 | 3.225 |  |
| **Hormone metabolism** |  |  |  |  |
| chr1.CM0033.30_at | PAL | -2.346 | n.r. |  |
| ljwgs_029302.1_x_at | PAL | 1.503 | n.r. |  |
| chr1.CM0033.36_at | PAL | 1.625 | n.r. |  |
| chr2.CM0191.90_at | PAL | 2.839 | n.r. |  |
| ljwgs_078032.1_at | PAL | 4.319 | 3.390 |  |
| ljwgs_018584.1_at | ICS | -2.259 | n.r. |  |
| chr2.CM1150.8_at | UGTase | 5.669 | 3.000 |  |
| chr2.CM0249.71.2_at | SAMT | 1.418 | n.r. |  |
| ljwgs_017855.1_x_at | SAMT | 4.130 | n.r. |  |
| chr1.CM0113.33_at | SAMT | 5.040 | 3.617 |  |
| ljwgs_099352.1_s_at | SAMT | 5.831 | n.r. |  |
| ljwgs_050995.1_s_at | SAMT | 6.227 | n.r. |  |
| ljwgs_020217.1_at | SAMT | 6.896 | n.r. |  |
| ljwgs_103120.1_at | PR-like | 1.304 | n.r. |  |
| chr1.TM1305.12_at | PR-like | 1.405 | n.r. |  |
| ljwgs_073106.1_at | PR-like | 1.428 | n.r. |  |
| ljwgs_063269.1_at | PR-like | 1.658 | n.r. |  |
| ljwgs_032901.1_s_at | PR-like | 2.151 | n.r. |  |
| ljwgs_026920.1_at | PR-like | 2.697 | 2.594 |  |
| ljwgs_027615.2_at | PR-like | 3.635 | n.r. |  |
| ljwgs_078238.1_at | PR-like | 3.872 | n.r. |  |
| ljwgs_020594.1_at | PR-like | 4.365 | 5.271 |  |
| chr1.CM0012.67_at | PR-like | 5.523 | 4.943 |  |
| ljwgs_115315.1_at | PR-like | 6.062 | n.r. |  |
| ljwgs_079986.1_at | PR-like | 6.242 | 5.197 |  |
| ljwgs_010724.1_s_at | NPR3-like | -2.245 | n.r. |  |
| chr3.CM0216.81_at | EDS5 | -1.478 | n.r. |  |
| ljwgs_048005.1_at | EDS1 | n.r. | n.r. | 1.858 |
| chr5.CM0096.73_at | LOX | -1.198 | n.r. |  |
| ljwgs_078570.2_at | LOX | 1.287 | n.r. |  |
| ljwgs_027386.1_at | LOX | 1.832 | n.r. |  |
| ljwgs_038042.1_s_at | LOX | 2.779 | n.r. |  |
| ljwgs_096797.1_s_at | LOX | 5.910 | n.r. |  |
| chr3.CM0115.22_at | LOX | n.r. | -1.560 | 3.321 |
| ljwgs_038744.1_s_at | LOX | n.r. | n.r. | 2.440 |
| ljwgs_134865.1_at | LOX | n.r. | n.r. | 2.651 |
| chr4.cm0042.112_at | Pathogen inducible α-dioxygenase | 1.628 | n.r. |  |
| ljwgs_093636.1_at | Pathogen inducible α-dioxygenase | 2.592 | n.r. | 2.644 |
| ljwgs_132514.1_s_at | AOS | 2.010 | n.r. |  |
| chr5.CM0089.88_at | AOS | 2.178 | n.r. |  |
| chr6.CM0314.12_s_at | AOS | 5.127 | n.r. |  |
| ljwgs_019853.2_at | AOC | 3.602 | n.r. |  |
| chr1.CM0591.20_at | OPR3 | -1.224 | n.r. |  |
| chr1.CM0591.12_at | OPR3 | 4.981 | n.r. |  |
| TM0763.11_at | OPR3 | 6.665 | 4.337 |  |
| chr1.CM0579.32_at | JAR1-like | -1.977 | n.r. |  |
| chr4.CM0042.119_at | JAR1-like | -1.542 | n.r. |  |
| ljwgs_091498.1_at | COI1-like | -1.699 | n.r. |  |
| chr4.CM0501.53_at | ACS | 1.856 | n.r. |  |
| ljwgs_033819.1_at | ACS | 3.306 | n.r. |  |
| chr1.TM1635.18_at | ACS | 6.937 | 5.104 |  |
| ljwgs_094520.1_at | ACO | 2.766 | n.r. |  |
| ljwgs_026218.1_at | ACO | 3.334 | n.r. |  |
| ljwgs_126357.1_at | ACO | 4.195 | n.r. |  |
| chr1.TM1666.4.1_at | ACO | 4.963 | n.r. |  |
| ljwgs_125699.1_at | ACO | 6.853 | n.r. |  |
| ljwgs_059500.1_at | ZEP | -3.125 | n.r. |  |
| ljwgs_024159.1_at | ZEP | -2.369 | n.r. |  |
| ljwgs_031176.1_at | ZEP | -2.195 | n.r. |  |
| chr3.TM0436.18_at | ZEP | -1.901 | n.r. |  |
| ljwgs_013491.1.1_at | NCED | -3.493 | n.r. |  |
| ljwgs_040068.1_at | SDR | -1.835 | n.r. |  |
| chr2.CM0545.2.4_at | AAO | -1.490 | n.r. |  |
| chr2.CM0545.8.2_at | AAO | -1.464 | n.r. |  |
| chr2.CM0124.37_at | GH3 | 1.673 | n.r. |  |
| ljwgs_030396.2_s_at | GH3 | 1.866 | n.r. |  |
| ljwgs_019565.1_at | GH3 | 3.157 | 2.618 |  |
| chr2.CM0250.19_at | GH3 | 4.485 | 2.907 |  |
| chr1.TM0207.17_at | IAA-AA hydrolase | -1.540 | n.r. |  |
| ljwgs_107420.1_s_at | IAA-AA hydrolase | -1.294 | n.r. |  |
| ljwgs_060317.1_s_at | IAA-AA hydrolase | -1.147 | n.r. |  |
| ljwgs_063190.1_s_at | IAA-AA hydrolase | 1.622 | n.r. |  |
| **Phenylpropanoids** |  |  |  |  |
| ljwgs_031602.1_at | C4H | 1.539 | 1.057 |  |
| ljwgs_082453.1_at | C4H | 4.476 | 2.719 |  |
| chr2.CM0020.32_at | 4CL | n.r. | n.r. | 1.225 |
| ljwgs_075474.1_at | 4CL | n.r. | n.r. | 1.460 |
| chr1.CM0591.42_x_at | CHS | 1.912 | n.r. |  |
| chr2.CM0018.54_x_at | CHS | 2.372 | n.r. |  |
| chr2.CM0018.90.1_at | CHS | 2.379 | n.r. |  |
| chr3.CM0590.56_at | CHS | 3.018 | n.r. |  |
| ljwgs_072307.1_at | CHS | 3.342 | 2.351 |  |
| ljwgs_012789.1_at | CHS | 3.354 | 1.834 |  |
| ljwgs_033436.3_x_at | DFR | 3.629 | n.r. | 3.141 |
| chr5.CM0077.20_at | DFR | 3.796 | 1.973 | 1.850 |
| chr2.CM0249.88_s_at | IFR | 1.149 | n.r. |  |
| chr3.CM0216.52_at | IFR | 2.302 | n.r. |  |
| ljwgs_020489.1_at | IFR | 2.764 | n.r. |  |
| chr3.CM0216.28_at | IFR | 3.425 | n.r. |  |
| ljwgs_031126.1_at | IFR | 4.614 | n.r. |  |
| chr2.CM0020.42.1_at | IFR | 6.054 | 3.593 |  |
| chr1.CM1255.32_at | VR | 1.887 | n.r. | 1.468 |
| chr1.CM1255.30_at | VR | 3.385 | n.r. | 2.385 |
| ljwgs_025931.2_s_at | F3H | -3.147 | n.r. |  |
| chr4.CM0119.36_at | F3H | -2.578 | n.r. |  |
| ljwgs_024122.1_at | F3H | -2.973 | n.r. |  |
| ljwgs_039470.1_at | F3H | -2.390 | n.r. |  |
| ljwgs_064158.1_at | F3H | -2.016 | n.r. |  |
| chr4.CM0042.77.1_at | CAD | -2.306 | n.r. |  |
| chr2.CM0177.18_at | CAD | 3.115 | n.r. |  |
| ljwgs_021748.1.1_at | CCR | 3.603 | n.r. |  |
| ljwgs_013806.1_at | COMT | -2.112 | n.r. |  |
| ljwgs_030453.1_at | COMT | 2.055 | n.r. | 1.225 |
| ljwgs_061978.2_at | COMT | 2.127 | n.r. | 2.007 |
| ljwgs_017175.2_at | COMT | 2.133 | n.r. | 2.342 |
| TM0623.12_at | COMT | 2.299 | n.r. |  |
| ljwgs_046265.1_at | COMT | 2.554 | n.r. | 1.427 |
| chr2.CM0191.69_at | COMT | 3.061 | 2.448 |  |
| **Abiotic stress** |  |  |  |  |
| ljwgs_126977.1_at | UV-damaged DNA binding | n.r. | n.r. | 1.331 |
| ljwgs_016038.1_at | Universal stress protein | n.r. | n.r. | 2.162 |
| ljwgs_081400.1_s_at | UV-damaged DNA binding | n.r. | n.r. | 6.036 |
