# Supplementary material for: Defense Responses in Two Ecotypes of Lotus japonicus against Non-Pathogenic Pseudomonas syringae
Source: PLoS One. 2013 Dec 11;8(12):e83199. doi: 10.1371/journal.pone.0083199 (PMC3859661; doi:10.1371/journal.pone.0083199)
Supplement: Table S3 — Microarray and qRT-PCR expression values for genes used to build Figure 5. (DOC) [file pone.0083199.s003.doc]

**Table S3. Microarray and qRT-PCR expression values for genes used to build Figure 5.**

| **Ecotype** | **Lotus Affymetrix ID** | **Gene annotation** | **Microarray data**  **(log_2_ mean relative**  **expression)** | **p value for microarray** | **qRT-PCR data**  **(log_2_ mean relative**  **expression)** | **p value for qRT-PCR** |
| --- | --- | --- | --- | --- | --- | --- |
| Gifu B-129 | ljwgs_090338.1_at | chr6.CM0013.530.r2.m | 2.232 | 0.002056 | 2.348 | 0.012 |
| Gifu B-129 | ljwgs_061086.1.1_at | LjSGA_045519.1 | 2.489 | 0.003094 | 3.013 | 0.025 |
| Gifu B-129 | chr1.bm1732.2_at | chr1.ljb18k24.110.r2.a | 4.144 | 0.000750 | 3.589 | 0.007 |
| Gifu B-129 | ljwgs_020594.1_at | chr4.CM1622.120.r2.d | 5.271 | 0.000502 | 4.648 | 0.009 |
| Gifu B-129 | ljwgs_013445.2_at | LjSGA_013445.2 | 1.311 | 0.031752 | -0.014 | 0.0471 |
| Gifu B-129 | ljwgs_086126.1_at | chr1.cm0295.1210.r2.a | 2.232 | 0.002056 | 2.348 | 0.012 |
| MG-20 | chr6.cm0539.8_at | chr6.cm0139.1430.r2.d | -2.521 | 0.016829 | -5.059 | 0.011 |
| MG-20 | ljwgs_025735.1_at | LjSGA_025735.1 | -1.563 | 0.025916 | -3.184 | 0.033 |
| MG-20 | chr3.cm0279.2_at | chr3.cm0279.1210.r2.d | 2.237 | 0.004366 | -1.786 | 0.019 |
| MG-20 | cm0528.2_at | chr4.cm0528.420.r2.d | -1.386 | 0.001566 | -0.811 | 0.0178 |
| MG-20 | ljwgs_068360. _at | chr5.cm1077.690.r2.m | 2.251 | 0.009972 | 2.046 | 0.046 |
| MG-20 | chr5.cm0953.1_at | chr5.CM0200.390.r2.d | 2.535 | 0.006791 | 2.307 | 0.011 |
| MG-20 | ljwgs_011581.2_at | chr3.cm279.180.r2.d | 1.930 | 0.001833 | 2.934 | 0.0092 |
| MG-20 | chr1.bm1732.2_at | chr1.ljb18k24.110.r2.a | 6.276 | 0.000010 | 9.428 | 0.008 |
